# *Sphingomonas wittichii* Strain RW1 Genome-Wide Gene Expression Shifts in Response to Dioxins and Clay

**DOI:** 10.1371/journal.pone.0157008

**Published:** 2016-06-16

**Authors:** Benli Chai, Tamara V. Tsoi, Shoko Iwai, Cun Liu, Jordan A. Fish, Cheng Gu, Timothy A. Johnson, Gerben Zylstra, Brian J. Teppen, Hui Li, Syed A. Hashsham, Stephen A. Boyd, James R. Cole, James M. Tiedje

**Affiliations:** 1 Center for Microbial Ecology, Michigan State University, East Lansing, MI, United States of America; 2 Plant, Soil and Microbial Sciences, Michigan State University, East Lansing, MI, United States of America; 3 Civil and Environmental Engineering, Michigan State University, East Lansing, MI, United States of America; 4 School of the Environment, Nanjing University, Nanjing, China; 5 Department of Biochemistry & Microbiology, Rutgers University, New Brunswick, NJ, United States of America; University of Florida, UNITED STATES

## Abstract

*Sphingomonas wittichii* strain RW1 (RW1) is one of the few strains that can grow on dibenzo-*p*-dioxin (DD). We conducted a transcriptomic study of RW1 using RNA-Seq to outline transcriptional responses to DD, dibenzofuran (DF), and the smectite clay mineral saponite with succinate as carbon source. The ability to grow on DD is rare compared to growth on the chemically similar DF even though the same initial dioxygenase may be involved in oxidation of both substrates. Therefore, we hypothesized the reason for this lies beyond catabolic pathways and may concern genes involved in processes for cell-substrate interactions such as substrate recognition, transport, and detoxification. Compared to succinate (SUC) as control carbon source, DF caused over 240 protein-coding genes to be differentially expressed, whereas more than 300 were differentially expressed with DD. Stress response genes were up-regulated in response to both DD and DF. This effect was stronger with DD than DF, suggesting a higher toxicity of DD compared to DF. Both DD and DF caused changes in expression of genes involved in active cross-membrane transport such as TonB-dependent receptor proteins, but the patterns of change differed between the two substrates. Multiple transcription factor genes also displayed expression patterns distinct to DD and DF growth. DD and DF induced the catechol ortho- and the salicylate/gentisate pathways, respectively. Both DD and DF induced the shared down-stream aliphatic intermediate compound pathway. Clay caused category-wide down-regulation of genes for cell motility and chemotaxis, particularly those involved in the synthesis, assembly and functioning of flagella. This is an environmentally important finding because clay is a major component of soil microbes’ microenvironment influencing local chemistry and may serve as a geosorbent for toxic pollutants. Similar to clay, DD and DF also affected expression of genes involved in motility and chemotaxis.

## Introduction

Polychlorinated dibenzo-*p*-dioxins and dibenzofurans (PCDD/PCDF) belong to a group of structurally related chemicals collectively called dioxins [1]. PCDD/PCDF are among the most environmentally persistent organic pollutants, and are known for their carcinogenic effects on humans [2,3]. Microbiologically mediated biodegradation represents a promising approach for the remediation of PCDD/PCDF contaminated soils and sediments. Extensive efforts have been expended on isolating (or attempting to isolate) bacteria and/or bacterial communities that can detoxify PCDD/PCDFs in natural environments. Several bacterial strains have been isolated that can grow on DF as sole carbon source or co-metabolize some PCDD/PCDF congeners in resting cell assays [3,1]. However, the ability to use DD as sole carbon source has been reported for only a few strains including *Sphingomonas wittichii* strain RW1 [4,5,3], which, to our knowledge, is the only one with a sequenced genome.

*Sphingomonas wittichii* strain RW1 is a member of the Alphaproteobacteria and was isolated from the Elbe River in Germany by its growth on DD [4]. It also grows on DF and 4-chloro-dibenzofuran [4,6,7,3].

The initial oxygenation of DD and DF by RW1 is catalyzed by an angular dioxin dioxygenase [8]. Meta-cleavage follows involving 2,2',3-trihydroxybiphenyl dioxygenase [9], and the hydrolysis of 2-hydroxy-6-oxo-6-(2-hydroxyphenyl)-hexa-2,4-dienoate (HOHPDA) by HOPDA hydrolase [4,10]. This process comprises the common upper pathway and produces salicylate from DF or catechol from DD. Both compounds are then further catabolized to aliphatic compounds through the gentisate or catechol ortho-pathways prior to entering TCA cycle for complete oxidation [4]. Genes coding for the upper pathway have been cloned and expressed in *E*. *coli* and mapped to the *dxn* region [11,12,13,14]. These studies show that most of the genes might be involved in oxidation of both DD and DF, but the role of the meta-cleavage oxygenase DbfB in DD metabolism was not confirmed. Very little activity against the DD hydroxylation product 2,2’,3-trihyroxydiphenylether occurred in DbfB-expressing *E*. *coli* recombinant cells [11]. Pathways for degradation of aliphatic compounds derived from the hydroxylated ring of dioxins have not been systematically studied.

The completion of the genome sequence of RW1 has greatly facilitated studies of its unique degradation capability. The RW1 genome consists of one chromosome and two mega plasmids [15]. One of the characteristics of the RW1 genome is the presence of multiple TonB-dependent outer membrane receptor protein genes. A transcriptomics study of RW1 grown on DF was reported [16] using a whole genome DNA microarray and transposon mutagenesis, and two proteomics studies examined the protein profiles of RW1 cells grown on DF [17] and when grown on DD, DF and DF in the presence of 2-chlorodibenzo-*p*-dioxin [18].

Although RW1 is able to utilize both DD and DF as its sole source of carbon, how differently RW1 responds to DD or DF is unknown. We hypothesized that the difficulty in isolating DD-growing bacteria compared with the relative ease in isolating DF-growing bacteria may reflect certain common metabolic constraints. Changes in the expression of genes outside of catabolic pathways may be required to allow RW1 to grow on DD. The whole transcriptome shotgun sequencing of RW1 grown on DD, DF, and SUC (control) enabled us to outline the overall differences in gene expression in response to DD and DF, as well as to confirm genes involved in DD and DF catabolism. Furthermore, in nature, poorly water-soluble compounds such as PCDD/PCDF associate extensively with geosorbents such as clays, organic matter, and chars [19,20]. Our prior work showed Cs-saponite clay to be an effective sorbent for DD, 10 to 50 times more adsorbent than Ca-, Mg- or K-saponite [20]. Hence, the effects of a major soil component, *viz*. clay, on global gene expression was also evaluated using Cs-saponite.

## Materials and Methods

*Sphingomonas wittichii* strain RW1 was kindly provided by Dr. Rolf Halden at Arizona State University. Genome sequences of RW1, chromosomal (NC_00911, open reading frames Swit_0001 to Swit_4886), plasmid pSWIT01 (NC_009507, Swit_5113 to Swit_5400) and plasmid pSWIT02 (NC_00908, Swit_4887 to Swit_5112), and annotations were downloaded from the Joint Genome Institute (JGI, http://genome.jgi-psf.org/sphwi/sphwi.home.html). Pathway information for RW1 was obtained from the Kyoto Encyclopedia of Genes and Genomes (KEGG, http://www.kegg.jp/kegg/).

To update the annotation of 172 differentially expressed open reading frames marked “hypothetical”, we conducted homology searches using BLASTP against the NCBI non-redundant protein database (nr, GenBank release 211) using cutoffs e-value ≤ 10^−10^ and aligned length ≥ 50%. Of 172 open reading frames searched, 17 produced hits to known genes.

### Clay preparation

Smectite clay (saponite, SapCa-2) obtained from the Source Clays Repository of the Clay Minerals Society at Purdue University was used in this study. Preparation of homoionic Cs-saponite followed the method of Arroyo et al. [21]. Briefly, a dilute clay suspension was titrated to pH 6.8 with 0.6 M sodium acetate buffer (pH = 5) to remove carbonate impurities. The clay-sized particles were obtained by centrifugation of the clay suspension for 6 min at 60×*g*, then treated with 0.1 M CsCl six times to saturate the cation exchange capacity of the clay with Cs^+^. The homoionic Cs-saponite was washed using Milli-Q water until free of chloride ion as indicated by failure to form AgCl precipitate when the wash water was mixed with AgNO_3_. The preparation was then freeze dried.

### Culturing, substrates, and clay

RW1 was grown at 30°C using defined mineral DSMZ medium 457 (Brunner medium) (Deutsche Sammlung von Mikroorganismen und Zellkulturen GmbH, http://www.dsmz.de/). Carbon substrates were added as follows: SUC was added to a final 20 mM concentration. DD and DF were added to sterile flasks from acetone stocks to a final nominal concentration of 3 mM and left open in a sterile hood for 5 h to allow acetone to completely evaporate. Brunner medium was then added and flasks were sonicated for 10 s to dislodge and break up substrate crystals. Cs-saponite was added to 0.7% (w/v) final concentration in Brunner medium with succinate and sonicated to obtain a suspension with homogenized clay particles (SAP cultures).

Cell density of cultures (CFU, colony forming units) was determined by plating serial dilutions on nutrient agar from the control succinate-only (SUC) cultures, the SAP cultures, and on Brunner agar with DF crystals (added onto Petri dish lid) from DD and DF cultures.

Scanning Electron Microscopy (SEM) was performed at MSU Center for Advanced Microscopy by Carol Flegler.

### RNA isolation and mRNA enrichment

Total RNA was isolated from early-to-mid log phase cultures (CFU of 1.0x10^9^ to 1.5x10^9^ cells/mL) using a PureLink Mini Kit (Life Technologies, Carlsbad, CA). To ensure complete removal of DNA, RNA samples underwent a two-step DNAse I treatment, first with DNAse I (amplification grade, Life Technologies) followed by TURBO DNAse I (Life Technologies). The RNA concentration was measured using Qubit (Life Technologies) and its quality was validated using BioAnalyzer (Agilent Technologies, Santa Clara, CA).

Cultures were sampled for transcriptomic analysis as follows: two replicates from SUC early- to mid-log phase (20–24 h), three from SAP early- to mid-log phase (33–38 h), three from DD early- to mid-log phase (47 h), and three from DF early- to mid-log phase (16 h). Enrichment of mRNA was performed by oligo-based removal of ribosomal RNA. A MicrobExpress kit (Life Technologies) was used for one SUC replicate and one SAP replicate. All other samples were enriched using a RiboMinus kit (Life Technologies) combined with custom oligonucleotides specifically targeting RW1 23S rRNA.

Designing the customized oligos was aided by Robin Gutell’s 23S rRNA secondary structure model diagram of *E*.*coli* (http://www.rna.icmb.utexas.edu/). Regions of 23S rRNA free of hairpin structures were targeted, and candidate oligo probes of 20–22 bp long were selected. These sequences were then used to search the RW1 genome sequence to filter out those sharing four or more consecutive identical bases with non-23S regions. Five oligo-probes were eventually selected and synthesized with poly(A) tails added at the 3’ end. The 23S-targeting oligos with poly(A) tail were used along with poly(T) coated Dynabeads (Life Technologies) for the removal of 23S rRNA through specific hybridization (using RiboMinus hybridization buffer).

### Illumina sequencing and data processing

Transcriptomic data consisting of 35-bp single-end reads was generated using an Illumina GA II system. Library preparation and sequencing were performed at the MSU Research Technology Support Facility. Sequence reads were filtered using the Illumina chastity filter. Reads were then trimmed using a custom Python script to remove the 3’ end low quality segments flagged by *Read Segment Quality Control Indicator*. Reads from each sample were aligned using Bowtie [22] to the RW1 genome reference sequences, from which the Bowtie index file was built, using the mapping parameters (*-n 2 -e 70 -l 28—best*). Only reads mapping to unique genomic sites were tallied and summarized in the count table using a custom Python script and the RW1 gene feature table from JGI. Read counts of technical replicates were pooled as they showed excellent reproducibility as measured by Pearson correlation coefficients (R^2^) above 0.99 (not shown).

Comparisons of genes between treatments for differential expression were performed using the DESeq R package [23,24]. This package uses the negative binomial distribution for better modeling read count variability over the dynamic range between biological replicates, normalizes effective library sizes with a median count based method, which is less prone to the skewing effect by the highly differentially expressed genes, and computes differential expression tests with p values adjusted (P_adj_) for multiple testing with the Benjamini-Hochberg procedure for controlling the False Discovery Rate. A rate of 5% (0.05) was used as the cutoff for calling differential expression.

Custom Python scripts are available at https://github.com/chaibenl/RW1.

Sequencing and metadata files are available at NCBI Gene Expression Omnibus (GEO): accession GSE74831 (SRA SRP065972; BioProject PRJNA301621).

## Results

### Growth patterns under three culture conditions

When grown in the presence of clay (SAP), the RW1 growth phase was delayed, but the doubling time and final cell yield were similar to those of the control culture (SUC) (S1A and S1B Fig). SEM showed similar cell sizes (~1 μM) in both SAP and SUC cultures (S2A and S2B Fig). While RW1 cells in the SUC cultures showed moderate aggregation, only single or double (apparently dividing) cells were observed in the presence of SAP. Small clay particles appeared to associate with cell surfaces and individual cells were found associated with larger clay aggregates.

RW1 grown on DD (S1C Fig) normally transitioned to exponential growth after a short lag phase and reached a doubling time of 8 h. Some cultures grew in a biphasic growth pattern, characterized by an initial slow growth phase (to OD600 = 0.2–0.25), followed by a second more rapid growth phase. RW1 grew on DF (S1D Fig) with a doubling time of 5 h, shorter than on SUC (8 h) and DD (8 h). SEM revealed that both DF and DD growth promoted strong cell aggregation and increased amounts of extracellular material compared to SUC culture; cell colonization on DF and DD crystals was also observed.

### Gene expression differed little between SAP and SUC

**Fig 1:**
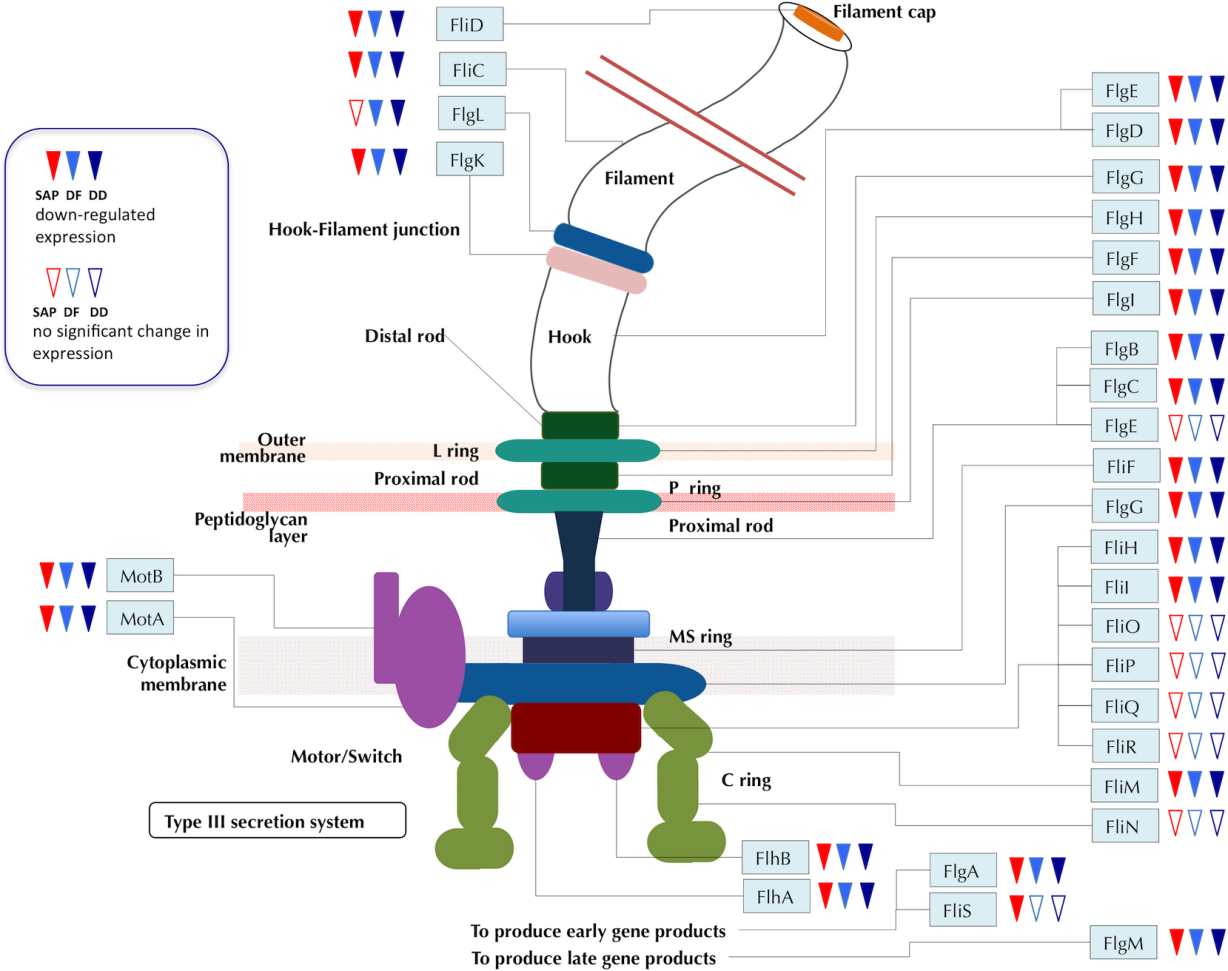
Flagellar Assembly: down-regulated genes compared to the SUC transcriptome.

Out of 5399 genes in RW1, we found 61 genes with statistically significant differential expression between the SAP-grown transcriptome and the SUC control. One gene was up-regulated while the remaining 60 were down-regulated in SAP, including genes for chemotaxis, signal transduction, pili, intracellular trafficking, and the majority of genes involved in flagellar assembly and functions (Fig 1).

### Differences in expression patterns under DD and DF compared to SUC

**Fig 2:**
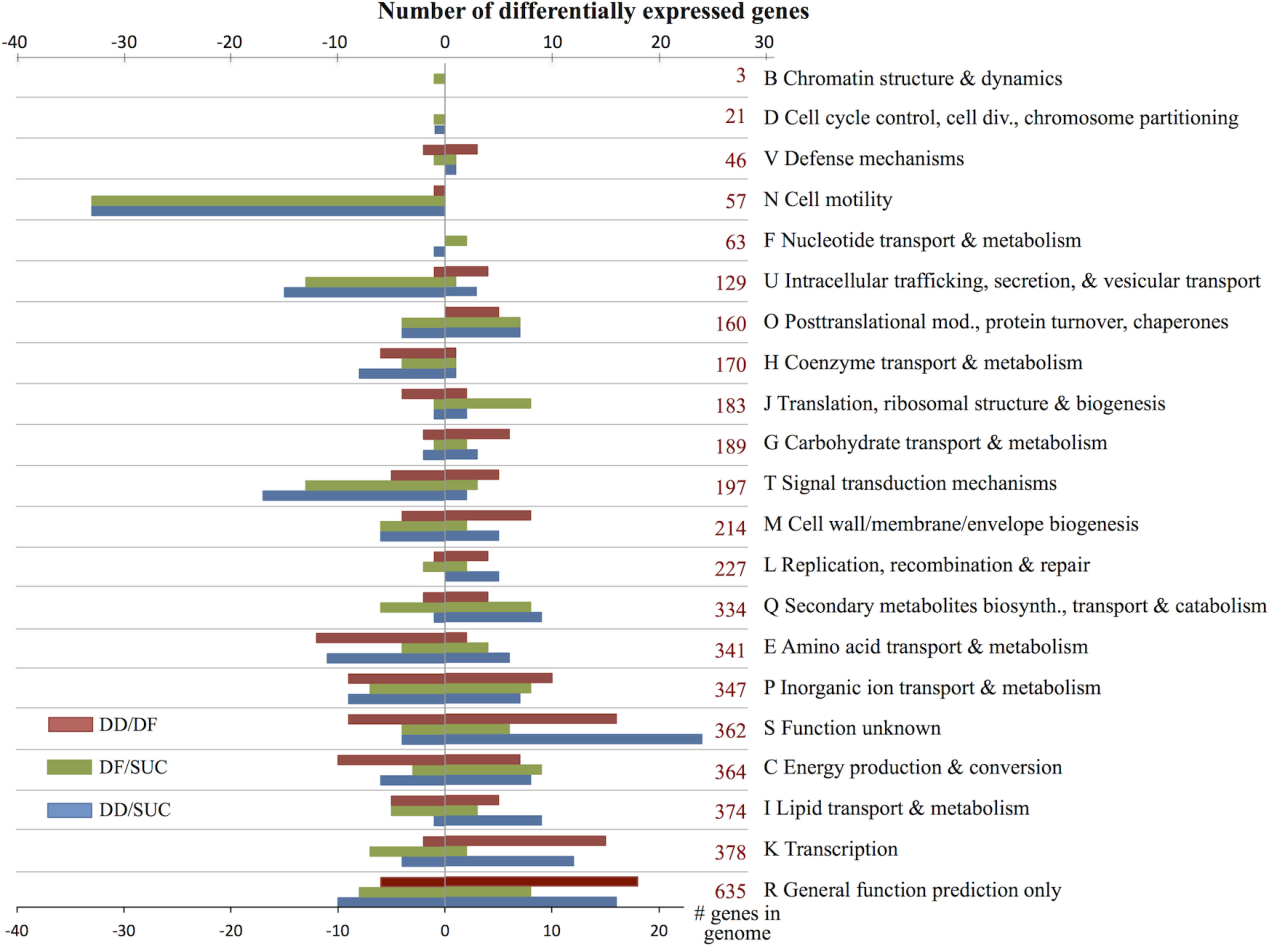
Numbers of up- and down-regulated genes, relative to SUC, in DD and DF grown cells, and relative to DF, in DD grown cells, by COG category. The numbers in red are total genes in each COG. Some genes are assigned to multiple COG categories and counted in each.

Relative to the SUC control, 320 and 241 genes were differentially expressed in response to DD and DF growth, respectively (Fig 2, S1 Table). The differentially expressed genes were distributed among 21 COG categories. The percentages of differentially expressed genes in each COG were not proportional to its relative abundance in the genome. DD and DF grown cells shared 35 up-regulated genes and 86 down-regulated genes. Only one of these shared genes was up-regulated in DD but down-regulated in DF, corresponding to a highly-expressed, conserved, hypothetical protein gene of only 141 nucleotides (Swit_4546).

For COGs D, N, F, U, O, T and Q, there were more differentially expressed genes in DD relative to SUC *and* more in DF relative to SUC than the number of differentially expressed genes in DD relative to DF (Fig 2, S2 Table). In particular, 30 genes in COG N were significantly down-regulated in response to both DD and DF while another three genes trended down in both comparisons but the difference was only significant in DD relative to SUC or DF relative to SUC, but not both (Fig 1, S2 Table). Only one COG N gene was significantly differentially expressed between DD and DF conditions, the methyl-accepting chemotaxis sensory transducer Swit_2932.

### Differential expression between DD and DF transcriptomes

Three hundred five genes in RW1 were significantly differentially expressed between DD and DF growth conditions, of which 136 did not show statistically significant differential expression between both DD and SUC and between DF and SUC (S1 Table). One hundred of the 305 genes differentially expressed between DD and DF are co-localized in 18 clusters containing 3 to 21 genes. These include a cluster of seven genes, with four significantly up-regulated in DD vs. DF. These are: two genes involved in malonate transport and genes for two subunits of malonate decarboxylase, two genes of unknown function, an acetyl-CoA carboxylase beta subunit-like protein (Swit_4284–4290), a cluster of pili and conjugation related genes on plasmid pSWIT01, (Swit_5364–5370), and two clusters encoding mainly hypothetical proteins, most of which are significantly up-regulated in DD compared to DF (Swit_2170–2188, and Swit_4460–4466). Overall, there were a total of 108 hypothetical protein-coding genes differentially expressed between DD and DF, of which 94 were up-regulated in DD relative to DF.

Our homology search of the current protein databases showed that 17 of the hypothetical protein genes are closely related to other annotated sequences in the databases (S1 Table). Most notable were genes encoding proteins related to a general stress protein (Swit_0545), a plasmid stabilization protein (Swit_0071), a conjugal transfer protein (Swit_5367), and a thiol:disulfide interchange protein (Swit_5369), all of which were significantly up-regulated in DD compared to both SUC and DF but not in DF compared to SUC. Another gene encodes a cytochrome C (Swit_4367), which was significantly up-regulated in DF compared to SUC and in DD compared to DF.

### Expression of DD and DF catabolic pathways

**Fig 3:**
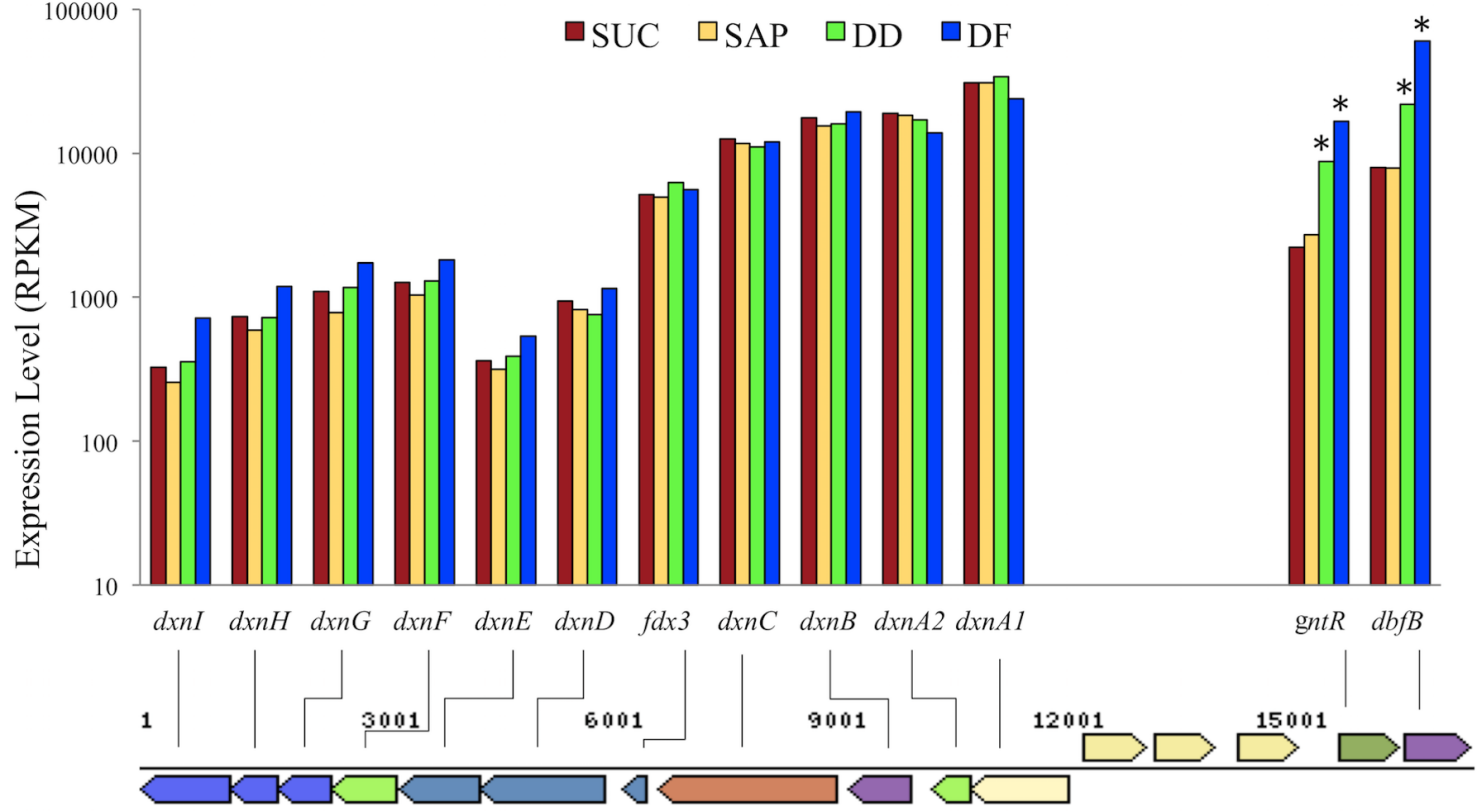
Organization and expression levels (RPKM counts) of dxn and *dbfB* genes. Dxn genes did not show statistically significant differential expression, while *dbfB* and *gntR* were induced by both dibenzo-*p*-dioxin (DD) and dibenzofuran (DF). * Indicates a significant difference (P_adj_-value ≤ 0.05) between SUC and both DD and DF. The differences between DD and DF are also significant for *dbfB*. For comparison, the median expression level for all genes was 49, 48 and 46 for SUC, DD and DF, respectively.

The upper pathway produces salicylate and 2-oxopent-4-enoate from DF, and catechol and 2-hydroxymuconate from DD. Upper pathway genes (S3 Fig), in general, showed high levels of constitutive expression in all three culture conditions, SUC, DD, and DF (Fig 3). The only differentially expressed gene in the upper pathway was *dbfB* (Swit_4902), which was significantly up-regulated 2.8-fold with DD, and up-regulated an additional 2.7-fold with DF (S1 Table, Fig 3). The gene for a transcription regulator from the GntR family located immediately upstream of *dbfB* was also up-regulated 3.9- and 7.5-fold with DD and DF, respectively.

**Fig 4:**
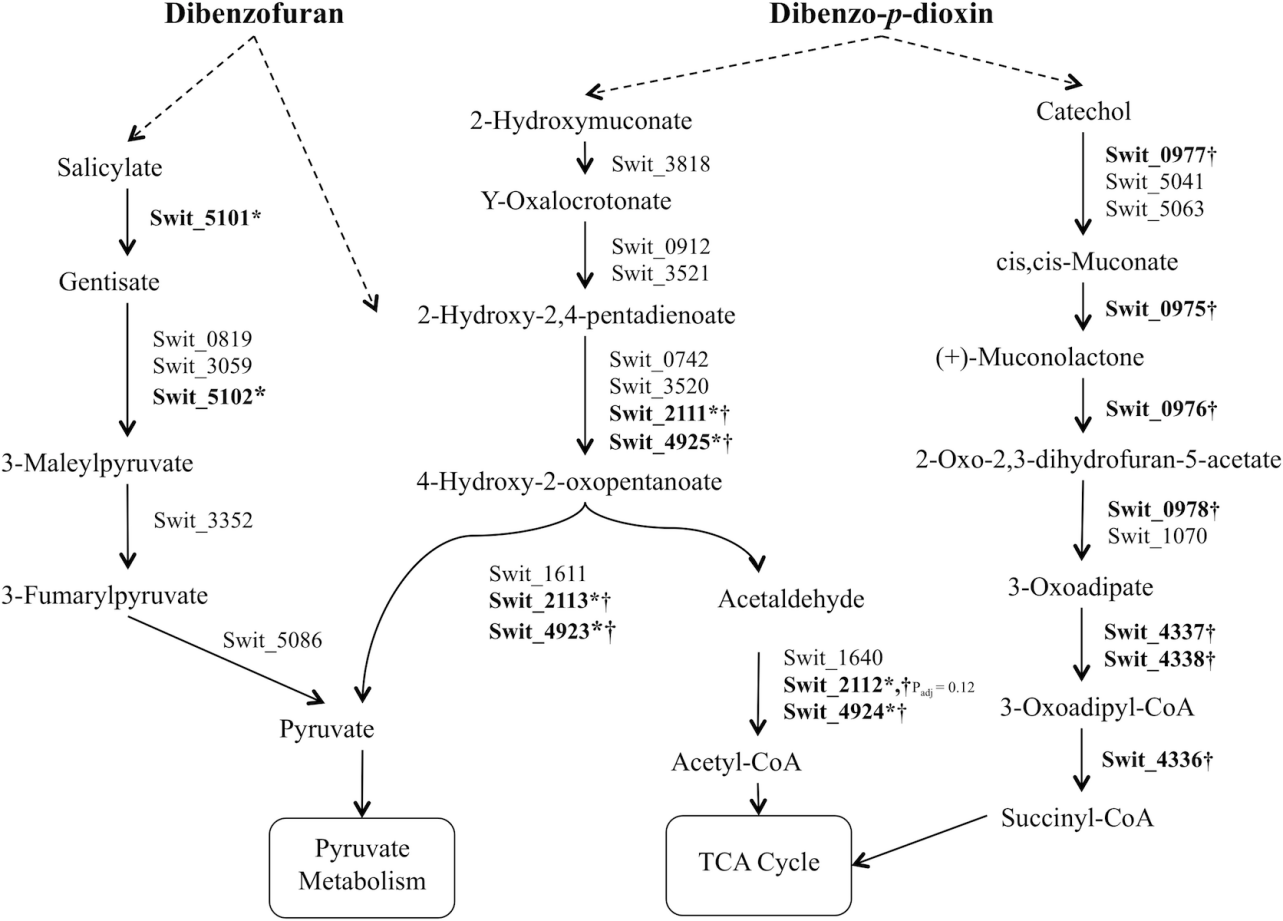
Lower pathways of dibenzo-*p*-dioxin and dibenzofuran degradation. Up-regulated genes are indicated in bold and * with DF and † with DD.

Compared to SUC, RW1 cultured with DD resulted in up-regulated genes for each step in the catechol *ortho*-pathway, which catalyzes the conversion of catechol to succinyl-CoA, while with DF two co-localized genes encoding salicylate 5-hydroxylase and gentisate 1,2 dioxygenase were up-regulated (Fig 4). In response to both DD and DF, key genes were significantly up-regulated in the aliphatic pathways through which 2-hydroxymuconate and 2-oxopent-4-enoate are catabolized to pyruvate and thereby pyruvate and acetyl-CoA entering the TCA cycle to complete degradation. These differentially expressed genes are located in two genome clusters (Swit_2111-Swit_2113, Swit_4923-Swit_4925), comprising three pairs of paralogs: 4-hydroxy 2-oxovalerate aldolase (EC:4.1.3.39) (Swit_2113, Swit_4923), which cleaves 4-hydroxy-2-oxopentanoate into acetaldehyde and pyruvate; acetaldehyde dehydrogenase (EC:1.2.1.10) (Swit_2112, Swit_4924), which catalyzes formation of acetyl-CoA from acetaldehyde; and 4-oxalocrotonate decarboxylase (EC:4.1.1.77) (Swit_2111, Swit_4925), which may participate in catalyzing decarboxylation of 4-oxalocrotonate derived from DD to form a common intermediate with DF, aliphatic product 2-oxopent-4-enoate, in further degradation.

## Discussion

We identified a large number of genes that responded similarly to DD and DF growth, which we expected since these are structurally similar compounds (S1 Table). The fact that many genes (14) in COG categories T and U were mostly down-regulated in response to both DD and DF may reflect the common suppressive effects of DD and DF to cell functions concerning signal transduction, intracellular trafficking, secretion, and vesicular transport.

Among known DF-degraders, RW1’s ability to grow on DD as its sole carbon source is rare. It also showed a significantly different growth pattern on DD compared to DF. We hypothesized that there would be some significant differences in gene expression, and that these differences would not be limited to genes in catabolic pathways but likely include those involved in processes for cell-substrate interactions such as substrate recognition, transport, and detoxification. Our findings largely confirmed this hypothesis since RW1 did show significant differences in gene expression in response to DD and DF (S1 Table). Most importantly, DD appeared to cause greater stress and apparent cytotoxicity responses than DF. This is exemplified by up-regulation of toxicity/stress response genes in DD vs. DF. These genes include an activator of HSP90 ATPase 1 family protein (Swit_1108), a general stress protein (Swit_0545), an entericidin EcnAB (Swit_3927), a glutathione-dependent formaldehyde-activating protein (Swit_4209), and glutathione S-transferases (Swit_0145, Swit_2245, Swit_3457), key enzymes that catalyze xenobiotic detoxification [25]. One cluster of efflux genes (Swit_1152, Swit_1153, Swit_1154) displayed a strong (18.1- to 27.8-fold increase) response to DD relative to DF. Genes for a Ku-family protein likely involved in non-homologous end-joining DNA repair (Swit_3981) [26], and a σ-24 RNA polymerase subunit (RpoE, extracytoplasmic/extreme heat stress factor) (Swit_0176) were up-regulated in response to DD relative to DF. RpoE was reported to be involved in tolerance to stresses caused by low or high temperature, low pH, and ethanol in *Yersinia pseudotuberculosis* [27]; more specifically, cell envelope stress response in *E*.*coli* and *Vibrio parahaemolyticus* has been reported [28,29].

Nevertheless DF also caused some stress response as witnessed by up-regulation of heat shock protein genes (Swit_4377, Swit_5351), chaperonin genes *groS* (Swit_3375) and *groL* (Swit_3376), encoding chaperonins Cpn10 (GroES) and Cpn60 (GroEL), respectively, and one efflux cluster (Swit_1951, Swit_1952). No stress response was detected in SAP relative to SUC. Chaperone gene *dnaK* (Swit_1250) and heat shock protein gene *dnaJ* (Swit_5306) displayed up-regulation by both DD and DF. Interestingly, these genes were also found up-regulated in response to PEG8000 by Johnson et al. [30], suggesting their possible role in a common mechanism in response to non-permeating solutes.

Chlorinated dibenzo-*p*-dioxins are known to cause cytotoxicity via DNA, membrane, oxidative and protein damage in studies using reporter genes [31,32]. Our results are indicative of a toxicity response elicited by non-chlorinated DD. Our results further suggest that cytotoxicity effects of DD were more pronounced in the early culture stage as shown in the DD1 transcriptome (S2 Table) which was derived from the early slow growth phase of the biphasic growth of RW1 (S1C Fig). Several toxicity response genes were up-regulated in the DD1 transcriptome, including a glutathione S-transferase gene (Swit_0145), a glutathione-dependent formaldehyde-activating gene (Swit_1412), and an aldehyde dehydrogenase gene (Swit_0703). Two genes (Swit_4550, Swit_4730) involved in defense mechanisms were up-regulated 6- to 19-fold (S2 Table). Genes in the efflux cluster (Swit_1152–1154), which were up-regulated in response to DD relative to SUC, also showed elevated expression in DD1 compared to DD though not statistically significant (Data not shown).

DD-mediated toxicity may, in part, explain the observation that although several DF degraders have been isolated [3], to our knowledge, only two DD degraders have been reported [4,5]. It is possible that the differential response to DD (compared to DF) by stress/toxic response genes may be one of the underlying factors enabling RW1 to both successfully overcome toxicity of, and grow on, DD.

Thirteen TonB-dependent receptor genes (included in COGs P, H, M) showed significant differential expression between DD and DF (S1 Table). An additional nine were significantly differentially expressed between DD and SUC and/or DF and SUC but not between DD and DF. None of the TonB-dependent receptor genes were significantly differentially expressed in SAP vs. SUC.

TonB-dependent transport across bacterial membranes has been shown for iron, vitamin D, colicins, sideromycins and several phages [33]. Recently the list of substrates was expanded to include carbohydrates (e.g., maltodextrins and sucrose) [34,35]. Codoy et al. [36] reported involvement of the TonB system in *Pseudomonas’* tolerance to toluene and 4-hydroxybenzoate. Our results implicate substrate specificity of cross-membrane transport in growth on dibenzo-*p*-dioxin and dibenzofuran.

Four genes (Swit_0687, Swit_2477, Swit_4025, Swit_4781) were up-regulated in cultures grown on DF compared to cultures grown both on DD and SUC, implying that they may be DF-specific. Two genes (Swit_1066, Swit_3263) were up-regulated in cultures grown on DD compared to cultures grown both on DF and SUC, implying that they may be DD-specific. Genome sequences are available for three other related organisms (*Sphingomonas* sp. DC-6, IMG: 2585427984; *Sphingomonas* sp. YL-JM2C, IMG: 2554235291; and *Sphingomonas wittichii*, DP58 IMG: 2548876930). Together these three organisms plus RW1 form a very closely related and distinct phylogenetic group based on overall genome similarity (JGI Clique ID: 1526). The genomes for each of these three contain homologs with ≥ 99% protein identity to Swit_1066, Swit_3263, but only RW1 contains genes for the upper pathway. If these two *tonB* gene products play a significant role in DD transport or detoxification, it is apparently separate from the ability to metabolize DD.

A number of TonB-dependent receptor genes were found highly up-regulated in DF vs. phenylalanine in Coronado et al. [16]. Of these, two genes (Swit_0687, Swit_4781) were also strongly up-regulated in DF vs. SUC in our study. It has been reported that *dxnC* (Swit_4894) from the dxn operon was up-regulated in DF vs. phenylalanine [16] and in DD vs. acetate [18], and was highly expressed across all treatments in our study; its role in transport of DD and DF is inconclusive. Moreno-Forero and van der Meer [37] reported only one TonB-dependent receptor gene (Swit_0540) up-regulated in response to sand impregnated with DF compared to DF liquid batch culture. This gene was not found differentially expressed in our or other studies. This may reflect differences in experimental design and conditions.

Our finding supports the current outline of dioxin catabolic pathways generated by previous studies. The *dbfB* gene, coding for a key enzyme (2,2',3-trihydroxybiphenyl dioxygenase) in the upper degradation pathway showed high levels of expression for both aromatic substrates and was significantly up-regulated in response to DD and significantly up-regulated in response to DF compared to DD (2.8- and 7.6-fold compared to SUC, respectively). This result contrasts with the proteomic study by Hartmann and Armengaud [18], who reported no change of this protein level between DD and DF growth. The *dbfB* gene appeared to be co-regulated with an adjacent *gntR* gene (Swit_4901) (S1 Table), in agreement with the findings of Coronado et al. [16].

One striking finding is that most genes known for cell motility were down-regulated when RW1 was grown with SAP, DD or DF. Of the 61 genes differentially regulated between SAP and SUC, 35 are related to cell motility (S2 Table), including flagella synthesis, assembly and function and chemotaxis (Fig 1). Genes for pilin and pilus assembly were also down-regulated. Saponite, but not Cs^+^ ion, is the actual effector since a transcriptome of cells grown on CsCl plus succinate did not show down-regulation of these genes (data not shown). The flagellar-specific transcription factor, σ-28 (FliA/WhiG, Swit_1281), responsible for transcription for flagellar biosynthesis and chemotaxis genes, along with anti σ-28 (FlgM, Swit_1275), were also down-regulated. The cyclic dinucleotide *c*-di-GMP-mediated signal transduction system was reported to be involved in motility and surface adhesion [38]. Its down-regulation in DD, DF and somewhat in SAP may contribute to the general suppression of cell motility of RW1 in these conditions.

Flagellum-dependent motility and chemotaxis are essential in enabling bacteria to move towards (positive chemotaxis) or away from (negative chemotaxis) chemical attractants and for the colonization of solid surfaces [39,40,41,42,43]. Previous work found a decrease in the transcription of several flagellar related genes (mostly in the Swit_1260-Swit_1293 cluster) during growth in low water potential conditions (growth in damp sand [37] or in medium containing high NaCl concentrations or polyethylene glycol [30]), while results from a study tagging genes with insertion elements found that some of these genes (e.g., Swit_1270 and Swit_1286) are important for survival during growth in partially saturated sand [44].

It seems likely that the presence of the saponite clay suppressed cell motility-related processes. Little is known about bacterial responses to the solid surfaces of their environment. In the case of RW1, no cell attachment to the clay minerals was observed except for some small particles of clay apparently clinging to cells (S2B Fig) in the dilute clay suspensions used in this study; at the much lower moisture content typical of soils, extensive cell attachment would be expected. It is not evident why the loss of pili or flagellum-dependent motility would benefit RW1 in adapting to clay minerals in suspension. It is clear however that clay minerals profoundly affected bacterial genes associated with motility, attachment and chemotaxis.

Many of the same flagellar genes were also strongly down-regulated in cells grown on DD and DF (S3 Table, Fig 1). In the presence of DD and DF crystals, it seems reasonable that the flagellar apparatus would be discarded since the bacteria were clearly colonizing the solid surfaces (S2 Fig) and would not be expected to require motility.

Despite this apparent loss of motility, the RW1 cells grown on DD and DF did not show differential expression of the pilin genes that were down-regulated in SAP (Swit_0616, Swit_3506) (S3 Table, Fig 1). Pili in bacterial cells are responsible for conjugation, pilus-mediated twitching motility, chemotaxis and cell-aggregation, which are all involved in cell-to-cell and cell-to-environment interactions that may affect the cell’s ability to aggregate and colonize surfaces [45,46]. It appears that expression of these pilin genes is associated with all three culture conditions (SUC, DD, and DF) in which the bacteria were observed to be social (S2 Fig), but not when the bacteria were apparently solitary in the presence of clay minerals. Potentially, interaction with DD and DF crystals by RW1 may also be mediated by pili.

## Supporting Information

S1 Table Differentially expressed genes between SUC, DD, and DF. Statistically significant differences are in a green background for increased, red for decreased; differences that are not statistically significant are in grey background with a green font for an increase and a red font for a decrease.
(DOCX)

S2 Table Genes differentially expressed between DD, and the early slow growth phase culture DD1. Colors as in S1 Table.
(DOCX)

S3 Table Expression changes of genes involved in cell motility under SAP, DD, and DF relative to SUC. Down-regulated genes are marked in red background, genes without significant change are marked in tan background. (COG N: Cell motility, COG M: Cell wall/membrane/envelope biogenesis; COG O: Post-translational modification, protein turnover, chaperones; COG T: Signal transduction mechanisms; COG U: Intracellular trafficking, secretion, and vesicular transport)
(DOCX)

S1 Fig Growth curves of RW1 cell cultures used for transcriptomes. RW1 cell cultures growing on (A) succinate as sole carbon source; (B) succinate as sole carbon source and in the presence of 0.7% Cs-Saponite; (C) 3 mM dibenzo-*p*-dioxin as sole carbon; (D) 3 mM dibenzofuran as sole carbon source. (Note one replicate of DD had delayed, biphasic growth).
(TIFF)

S2 Fig Scanning Electron Microscopy (SEM) images of RW1 cells. (A) SUC—cells self-aggregate, produce expolysaccharide and slime; (B) SAP—cells appear free-floating and did not aggregate or produce slime, but were attracted to smaller clay particles; (C) DD and (D) DF—cells form large aggregates, attached to DF crystals. White bar is 100 μm. Inset shows larger image of same sample.
(TIFF)

S3 Fig Upper pathway of dibenzofuran/dibenzo-*p*-dioxin degradation. *dbfB* was up-regulated (*†) in response to both dibenzofuran (DF) and dibenzo-*p*-dioxin (DD).
(TIFF)
