# Supplementary material for: Sphingomonas wittichii Strain RW1 Genome-Wide Gene Expression Shifts in Response to Dioxins and Clay
Source: PLoS One. 2016 Jun 16;11(6):e0157008. doi: 10.1371/journal.pone.0157008 (PMC4911050; doi:10.1371/journal.pone.0157008)
Supplement: S1 Table — Statistically significant differences are in a green background for increased, red for decreased; differences that are not statistically significant are in grey background with a green font for an increase and a red font for a decrease. (DOCX) [file pone.0157008.s001.docx]

S1 Table. Differentially expressed genes between SUC, DD, and DF. Statistically significant differences are in a green background for increased, red for decreased; differences that are not statistically significant are in grey background with a green font for an increase and a red font for a decrease. * Gene categorized to more than one COGs. † additional annotated by authors.

| **Gene ID** | **Product** | **Fold Change**  **DD/SUC** | **Fold Change**  **DF/SUC** | **Fold Change**  **DD/DF** |
| --- | --- | --- | --- | --- |
| **B Chromatin structure and dynamics** | | | | |
| Swit_3915* | histone deacetylase superfamily | **2.6** | **6.1** | **2.3** |
|  |  |  |  |  |
| **C Energy production and conversion** | | | | |
| Swit_0684 | pseudoazurin | **1.8** | **9.7** | **5.2** |
| Swit_0691 | hypothetical protein | **2.3** | **5.4** | **2.3** |
| Swit_0703 | aldehyde dehydrogenase (EC:1.2.1.3) | **1.8** | **5.3** | **2.8** |
| Swit_1713 | L-carnitine dehydratase/bile acid-inducible protein F (EC:2.8.3.16) | **2.9** | **2.5** | **1.2** |
| Swit_2532 | Inorganic diphosphatase (EC:3.6.1.1) | **2.4** | **4.8** | **2.0** |
| Swit_2629 | sodium:dicarboxylate symporter | **4.0** | **2.7** | **1.5** |
| Swit_2772 | V-type H(+)-translocating pyrophosphatase (EC:3.6.1.1) | **4.1** | **3.4** | **1.2** |
| Swit_2860 | aldo/keto reductase | **3.3** | **1.5** | **2.1** |
| Swit_2926 | isocitrate lyase and phosphorylmutase (EC:4.1.3.1) | **35.1** | **3.0** | **11.7** |
| Swit_2959 | FAD-dependent pyridine nucleotide-disulphide oxidoreductase (EC:1.6.99.3) | **3.6** | **3.0** | **1.2** |
| Swit_3030 | pyruvate ferredoxin/flavodoxin oxidoreductase (EC:1.2.7.8) | **2.8** | **1.0** | **2.8** |
| Swit_3045* | monooxygenase, FAD-binding (EC:1.14.13.20) | **1.7** | **2.9** | **1.7** |
| Swit_3200 | lycopene cyclase (EC:1.14.-) | **1.1** | **2.4** | **2.5** |
| Swit_3240 | aldehyde dehydrogenase | **3.9** | **1.2** | **4.6** |
| Swit_3255 | cytochrome c, class I | **1.6** | **3.9** | **2.4** |
| Swit_3265 | ferredoxin (EC:1.14.13.82) | **7.6** | **1.2** | **6.5** |
| Swit_3838 | malate synthase G (EC:2.3.3.9) | **2.1** | **1.4** | **3.1** |
| Swit_3977 | (2Fe-2S)-binding domain protein (EC:1.3.99.16) | **2.8** | **1.2** | **2.4** |
| Swit_4201* | D-isomer specific 2-hydroxyacid dehydrogenase, NAD-binding | **6.5** | **1.9** | **3.4** |
| Swit_4323* | D-isomer specific 2-hydroxyacid dehydrogenase, NAD-binding | **3.8** | **1.8** | **2.1** |
| Swit_4486 | H+-transporting two-sector ATPase, B/B' subunit (EC:3.6.3.14) | **1.7** | **1.8** | **3.1** |
| Swit_5088 | ferredoxin (Fdx1) | **2.0** | **1.6** | **3.2** |
| Swit_5101* | monooxygenase, FAD-binding (Salicylate hydroxylase Coronado 2012) | **1.1** | **21.7** | **18.9** |
| Swit_5151 | dihydrolipoamide dehydrogenase (EC:1.8.1.4) | **2.7** | **9.1** | **3.4** |
| Swit_5152 | pyruvate dehydrogenase complex dihydrolipoamide acetyltransferase (EC:2.3.1.12) | **1.7** | **5.7** | **3.5** |
| Swit_5153 | Transketolase, central region (EC:1.2.4.1) | **3.2** | **9.3** | **2.9** |
| Swit_5154 | Pyruvate dehydrogenase (acetyl-transferring) (EC:1.2.4.1) | **4.5** | **10.8** | **2.4** |
| Swit_5203 | Nitric oxide dioxygenase | **2.0** | **2.8** | **5.4** |
| Swit_5313 | (2Fe-2S)-binding domain protein | **4.5** | **1.4** | **3.3** |
|  |  |  |  |  |
| **D Cell cycle control, cell division, chromosome partitioning** | | | | |
| Swit_1280 | Cobyrinic acid a,c-diamide synthase | **18.6** | **9.4** | **2.0** |
|  |  |  |  |  |
| **E Amino acid transport and metabolism** | | | | |
| Swit_0438* | carbamoyl-phosphate synthase, large subunit (EC:6.3.5.5) | **1.8** | **4.2** | **2.4** |
| Swit_0536 | amino acid permease-associated region | **4.7** | **4.3** | **1.1** |
| Swit_0692* | extracellular solute-binding protein, family 3 | **1.9** | **6.3** | **3.4** |
| Swit_1203 | Agmatine deiminase (EC:3.5.3.12) | **2.4** | **1.1** | **2.7** |
| Swit_2113 | pyruvate carboxyltransferase (EC:4.1.3.39) | **4.6** | **13.0** | **2.8** |
| Swit_2399 | methionine synthase (EC:2.1.1.13) | **1.9** | **2.0** | **3.7** |
| Swit_2400 | homocysteine S-methyltransferase (EC:2.1.1.13) | **2.8** | **1.1** | **3.1** |
| Swit_2401 | 5,10-methylenetetrahydrofolate reductase (EC:1.5.1.20) | **4.5** | **1.3** | **3.3** |
| Swit_2414 | peptidase S10, serine carboxypeptidase | **10.6** | **9.0** | **1.2** |
| Swit_2417 | peptidase S10, serine carboxypeptidase | **3.6** | **2.1** | **1.7** |
| Swit_2418 | amino acid permease-associated region | **2.6** | **4.0** | **1.5** |
| Swit_2419 | aminotransferase, class I and II | **2.7** | **4.4** | **1.6** |
| Swit_2696 | Glycine dehydrogenase (decarboxylating) (EC:1.4.4.2) | **3.2** | **1.2** | **2.7** |
| Swit_2697 | Glycine dehydrogenase (decarboxylating) (EC:1.4.4.2) | **3.4** | **1.2** | **2.8** |
| Swit_2720 | imidazoleglycerol phosphate synthase, cyclase subunit (EC:4.1.3.-) | **2.0** | **1.5** | **2.9** |
| Swit_2777 | ATP phosphoribosyltransferase (EC:2.4.2.17) | **4.4** | **1.4** | **3.0** |
| Swit_2866 | Glyoxalase/bleomycin resistance protein/dioxygenase | **3.8** | **2.2** | **1.8** |
| Swit_2911 | 2,3,4,5-tetrahydropyridine-2,6-dicarboxylate N-succinyltransferase (EC:2.3.1.117) | **1.2** | **2.2** | **2.6** |
| Swit_3585 | Serine O-acetyltransferase (EC:2.3.1.30) | **3.1** | **1.3** | **2.4** |
| Swit_4030 | Orn/DAP/Arg decarboxylase 2 (EC:4.1.1.20) | **3.2** | **2.4** | **1.3** |
| Swit_4541 | SAF domain | **2.3** | **3.0** | **7.0** |
| Swit_4548 | asparagine synthase (EC:6.3.5.4) | **2.3** | **1.5** | **3.5** |
| Swit_4632 | histidine ammonia-lyase (EC:4.3.1.3) | **2.8** | **2.0** | **1.4** |
| Swit_4670 | Choline dehydrogenase and related flavoprotein-like protein | **4.3** | **1.5** | **2.9** |
| Swit_4786 | 5-methyltetrahydropteroyltriglutamate-- homocysteine S-methyltransferase (EC:2.1.1.14) | **4.5** | **1.4** | **3.2** |
| Swit_4923 | pyruvate carboxyltransferase (EC:4.1.3.39) | **9.7** | **16.6** | **1.7** |
| Swit_5345* | Alcohol dehydrogenase GroES domain protein (EC:1.1.1.284) | **3.2** | **1.2** | **2.7** |
|  |  |  |  |  |
| **F Nucleotide transport and metabolism** | | | | |
| Swit_0438* | carbamoyl-phosphate synthase, large subunit (EC:6.3.5.5) | **1.8** | **4.2** | **2.4** |
| Swit_0603 | dihydroorotase, multifunctional complex type (EC:3.5.2.3) | **4.6** | **1.9** | **2.4** |
| Swit_1504 | ribonucleoside-diphosphate reductase, alpha subunit (EC:1.17.4.1) | **1.1** | **2.8** | **2.5** |
|  |  |  |  |  |
| **G Carbohydrate transport and metabolism** | | | | |
| Swit_0121 | PfkB domain protein | **2.5** | **1.1** | **2.7** |
| Swit_0552 | PEP phosphonomutase and related enzyme-like protein | **19.2** | **1.8** | **10.4** |
| Swit_0553 | major facilitator superfamily MFS_1 | **246.7** | **1.8** | **136.0** |
| Swit_0693 | Pyrrolo-quinoline quinone | **1.8** | **8.2** | **4.5** |
| Swit_0696 | hypothetical protein (**glycosidase †**) | **1.6** | **2.9** | **4.8** |
| Swit_0710 | major facilitator superfamily MFS_1 | **1.8** | **2.2** | **3.9** |
| Swit_1441 | Pyrrolo-quinoline quinone (EC:1.1.99.8) | **4.0** | **1.5** | **2.6** |
| Swit_1886 | Mannitol dehydrogenase, C-terminal domain (EC:1.1.1.57) | **5.4** | **1.3** | **4.1** |
| Swit_1902 | Xylose isomerase domain protein TIM barrel | **3.7** | **5.6** | **1.5** |
| Swit_4552 | glycoside hydrolase, family 16 | **1.5** | **2.2** | **3.4** |
| Swit_4922 | Pyruvate, phosphate dikinase | **5.2** | **9.8** | **1.9** |
| Swit_5288* | PEBP family protein | **2.3** | **1.3** | **2.8** |
|  |  |  |  |  |
| **H Coenzyme transport and metabolism** | | | | |
| Swit_0427 | ApbE family lipoprotein | **1.6** | **1.8** | **2.9** |
| Swit_2402* | Methyltransferase type 11 | **2.6** | **1.1** | **3.0** |
| Swit_2699 | 2-dehydropantoate 2-reductase (EC:1.1.1.169) | **6.2** | **5.9** | **1.1** |
| Swit_2729 | FolC bifunctional protein (EC:6.3.2.17,EC:6.3.2.12,EC:6.3.2.17) | **3.6** | **1.6** | **2.2** |
| Swit_2880 | amine oxidase | **3.8** | **6.0** | **1.6** |
| Swit_3045* | monooxygenase, FAD-binding (EC:1.14.13.20) | **1.7** | **2.9** | **1.7** |
| Swit_3144 | TonB-dependent receptor | **2.1** | **3.1** | **1.5** |
| Swit_3189 | TonB-dependent receptor, plug | **3.6** | **1.4** | **5.0** |
| Swit_3544 | Methyltransferase type 11 | **2.7** | **1.1** | **2.9** |
| Swit_4201* | D-isomer specific 2-hydroxyacid dehydrogenase, NAD-binding | **6.5** | **1.9** | **3.4** |
| Swit_4291 | Triphosphoribosyl-dephospho-CoA synthase (EC:2.7.8.25) | **6.7** | **1.6** | **4.2** |
| Swit_4323* | D-isomer specific 2-hydroxyacid dehydrogenase, NAD-binding | **3.8** | **1.8** | **2.1** |
| Swit_4785 | dihydroneopterin aldolase (EC:4.1.2.25) | **7.8** | **1.6** | **5.0** |
| Swit_5101* | monooxygenase, FAD-binding (Salicylate hydroxylase Coronado 2012) | **1.1** | **21.7** | **18.9** |
|  |  |  |  |  |
| **I Lipid transport and metabolism** | | | | |
| Swit_0088* | acyl carrier protein | **3.5** | **3.6** | **1.0** |
| Swit_0380* | short-chain dehydrogenase/reductase SDR (EC:1.1.1.69) | **2.1** | **7.4** | **3.6** |
| Swit_0688 | acetate--CoA ligase (EC:6.2.1.1) | **1.2** | **3.3** | **4.0** |
| Swit_0769* | short-chain dehydrogenase/reductase SDR | **1.8** | **6.4** | **3.6** |
| Swit_0958 | 3-oxoacid CoA-transferase, B subunit (EC:2.8.3.5) | **5.0** | **2.2** | **2.3** |
| Swit_0959 | 3-oxoacid CoA-transferase, A subunit (EC:2.8.3.5) | **6.6** | **2.3** | **2.8** |
| Swit_1836 | acyl-CoA dehydrogenase domain protein (EC:1.3.99.13,EC:1.3.99.3) | **1.0** | **4.6** | **4.7** |
| Swit_1850 | acyl-CoA dehydrogenase domain protein | **1.1** | **4.3** | **4.6** |
| Swit_2497 | Nucleotidyl transferase | **17.8** | **17.8** | **1.0** |
| Swit_2636* | short-chain dehydrogenase/reductase SDR (EC:1.3.1.25) | **2.3** | **5.5** | **2.3** |
| Swit_3274 | acyl-CoA dehydrogenase domain protein (EC:1.3.99.10) | **2.9** | **4.9** | **14.1** |
| Swit_3372 | Phospholipid N-methyltransferase-like protein | **2.9** | **1.0** | **3.0** |
| Swit_3629 | acyl-CoA dehydrogenase domain protein (EC:1.3.99.2,EC:1.3.99.3) | **2.2** | **10.5** | **4.9** |
| Swit_3903* | diacylglycerol kinase, catalytic region | **1.7** | **3.2** | **1.9** |
| Swit_4288 | Acetyl-CoA carboxylase beta subunit-like protein (EC:6.4.1.3) | **7.4** | **2.3** | **3.3** |
| Swit_4335* | short-chain dehydrogenase/reductase SDR (EC:1.1.1.100) | **13.1** | **1.3** | **17.4** |
| Swit_4336 | beta-ketoadipyl CoA thiolase (EC:2.3.1.174) | **5.5** | **1.0** | **5.5** |
| Swit_4337 | 3-oxoacid CoA-transferase, B subunit (EC:2.8.3.6) | **7.2** | **1.2** | **6.3** |
| Swit_4338 | 3-oxoacid CoA-transferase, A subunit (EC:2.8.3.6) | **12.4** | **1.7** | **7.3** |
| Swit_5291* | short-chain dehydrogenase/reductase SDR | **4.5** | **2.5** | **1.8** |
|  |  |  |  |  |
| **J Translation, ribosomal structure and biogenesis** | | | | |
| Swit_0061 | pseudouridine synthase, RluA family (EC:5.4.99.12) | **4.4** | **4.6** | **1.0** |
| Swit_0461 | translation elongation factor Ts | **1.7** | **1.8** | **3.0** |
| Swit_0567 | ribonuclease PH (EC:2.7.7.56) | **3.3** | **1.4** | **2.3** |
| Swit_0887 | Amidase; aspartyl-tRNA(Asn)/glutamyl-tRNA (Gln) amidotransferase subunit A (EC:6.3.5.6,EC:6.3.5.7) | **2.7** | **1.0** | **2.7** |
| Swit_0927 | Amidase (EC:3.5.1.4) | **4.0** | **5.4** | **1.4** |
| Swit_1335 | ribosomal protein L30 | **1.3** | **3.1** | **2.3** |
| Swit_1338 | ribosomal protein L6 | **1.4** | **3.5** | **2.5** |
| Swit_1340 | ribosomal protein S14 | **1.4** | **3.6** | **2.5** |
| Swit_1341 | ribosomal protein L5 | **1.6** | **3.6** | **2.3** |
| Swit_1344 | ribosomal protein S17 | **1.7** | **3.6** | **2.2** |
| Swit_1345 | ribosomal protein L29 | **1.3** | **3.0** | **2.4** |
| Swit_3476 | ribosomal protein L7/L12 | **1.6** | **4.5** | **2.8** |
| Swit_3477 | ribosomal protein L10 | **1.9** | **1.9** | **3.5** |
| Swit_3810 | Polyribonucleotide nucleotidyltransferase (EC:2.7.7.8) | **1.7** | **1.6** | **2.8** |
| Swit_4540* | glucose-1-phosphate cytidylyltransferase (EC:2.7.7.33) | **5.1** | **1.1** | **4.6** |
|  |  |  |  |  |
| **K Transcription** | | | | |
| Swit_0060 | RNA polymerase sigma factor RpoH | **4.8** | **2.0** | **2.5** |
| Swit_0097 | transcriptional regulator, MarR family | **2.8** | **1.3** | **3.6** |
| Swit_0172 | transcriptional regulator, XRE family | **4.2** | **2.3** | **1.8** |
| Swit_0175* | response regulator receiver protein | **2.3** | **1.7** | **3.8** |
| Swit_0176 | RNA polymerase, sigma-24 subunit, ECF subfamily | **4.5** | **1.5** | **3.0** |
| Swit_0833 | regulatory protein, LuxR | **1.3** | **2.9** | **2.2** |
| Swit_0881 | transcriptional regulator, PadR-like family | **4.9** | **1.6** | **3.1** |
| Swit_1123 | putative transcriptional regulator, MerR family | **1.9** | **1.8** | **3.5** |
| Swit_1281 | RNA polymerase, sigma 28 subunit, FliA/WhiG | **5.9** | **8.2** | **1.4** |
| Swit_1285* | sigma54 specific transcriptional regulator, Fis family | **6.4** | **5.9** | **1.1** |
| Swit_2039 | helix-turn-helix- domain containing protein, AraC type | **1.5** | **2.5** | **3.7** |
| Swit_2402* | Methyltransferase type 11 | **2.6** | **1.1** | **3.0** |
| Swit_2919 | transcriptional regulator, BadM/Rrf2 family | **1.1** | **3.8** | **3.6** |
| Swit_2967 | transcriptional regulator, TetR family | **3.2** | **1.0** | **3.1** |
| Swit_3042 | transcriptional regulator, MarR family | **1.8** | **3.2** | **1.8** |
| Swit_3187* | response regulator receiver protein | **4.2** | **6.0** | **1.4** |
| Swit_3503 | parB-like partition protein | **2.0** | **1.2** | **2.4** |
| Swit_3569 | transcriptional regulator, TetR family | **7.4** | **1.2** | **6.4** |
| Swit_4054 | transcriptional regulator, MarR family | **3.3** | **1.0** | **3.3** |
| Swit_4177 | helix-turn-helix- domain containing protein, AraC type | **1.6** | **2.4** | **3.8** |
| Swit_4347 | helix-turn-helix- domain containing protein, AraC type | **1.4** | **1.9** | **2.8** |
| Swit_4362 | transcriptional regulator, TetR family | **8.7** | **7.3** | **1.2** |
| Swit_4510 | transcriptional regulator, MarR family | **5.8** | **1.0** | **5.6** |
| Swit_4649 | transcriptional regulator, LysR family | **3.8** | **5.9** | **1.6** |
| Swit_4803 | helix-turn-helix- domain containing protein, AraC type | **1.3** | **2.3** | **3.1** |
| Swit_4901 | transcriptional regulator, GntR family | **3.9** | **7.5** | **1.9** |
| Swit_5012 | regulatory protein, LuxR | **5.9** | **3.1** | **1.9** |
| Swit_5090 | Pseudogene | **4.1** | **1.1** | **4.5** |
| Swit_5196 | parB-like partition protein | **1.9** | **3.4** | **1.8** |
|  |  |  |  |  |
| **L Replication, recombination and repair** | | | | |
| Swit_0001 | chromosomal replication initiator protein DnaA | **1.2** | **3.8** | **3.2** |
| Swit_0045 | histone family protein DNA-binding protein | **1.2** | **2.7** | **2.3** |
| Swit_0651 | Excinuclease ABC, C subunit domain protein | **4.2** | **3.9** | **1.1** |
| Swit_2199 | D12 class N6 adenine-specific DNA methyltransferase (EC:2.1.1.72) | **10.5** | **4.4** | **2.4** |
| Swit_2359 | Integrase, catalytic region | **5.6** | **2.0** | **2.7** |
| Swit_3384 | Resolvase, N-terminal domain | **29.6** | **5.6** | **5.3** |
| Swit_4982 | Integrase, catalytic region | **1.3** | **3.0** | **3.8** |
| Swit_5104 | IstB domain protein ATP-binding protein | **3.3** | **16.9** | **5.1** |
| Swit_5118 | addiction module antitoxin, RelB/DinJ family | **2.2** | **1.3** | **2.8** |
| Swit_5215 | histone family protein DNA-binding protein | **3.7** | **2.0** | **1.9** |
|  |  |  |  |  |
| **M Cell wall/membrane/envelope biogenesis** | | | | |
| Swit_0493 | Rod shape-determining protein MreC | **3.7** | **1.1** | **3.3** |
| Swit_0792 | DegT/DnrJ/EryC1/StrS aminotransferase | **10.3** | **6.9** | **1.5** |
| Swit_0975* | muconate and chloromuconate cycloisomerase (EC:5.5.1.1) | **27.5** | **2.0** | **13.8** |
| Swit_1152 | efflux transporter, RND family, MFP subunit | **29.7** | **1.6** | **18.7** |
| Swit_1154* | RND efflux system, outer membrane lipoprotein, NodT family | **50.0** | **1.8** | **27.8** |
| Swit_1234 | OmpA/MotB domain protein | **1.3** | **2.8** | **3.6** |
| Swit_1263* | Rod binding-like protein | **3.6** | **6.9** | **1.9** |
| Swit_1807 | glycosyl transferase, group 1 | **4.4** | **12.2** | **2.7** |
| Swit_1814 | glycosyl transferase, group 1 | **4.0** | **8.0** | **2.0** |
| Swit_1951* | RND efflux system, outer membrane lipoprotein, NodT family | **1.1** | **5.5** | **5.0** |
| Swit_2322 | OmpA/MotB domain protein | **3.0** | **1.3** | **2.4** |
| Swit_2346 | lipoprotein releasing system, transmembrane protein, LolC/E family | **1.4** | **1.9** | **2.6** |
| Swit_2433 | AsmA family protein | **2.8** | **1.2** | **3.3** |
| Swit_2441 | TonB family protein | **1.2** | **3.2** | **2.7** |
| Swit_2577 | glycosyl transferase, group 1 | **2.2** | **1.3** | **2.8** |
| Swit_3438 | Mannose-1-phosphate guanylyltransferase (GDP) (EC:2.7.7.22,EC:5.3.1.8) | **3.2** | **1.6** | **2.0** |
| Swit_4477 | KpsF/GutQ family protein (EC:5.3.1.13) | **3.5** | **7.8** | **2.2** |
| Swit_4531 | polysaccharide export protein | **3.0** | **1.1** | **3.2** |
| Swit_4540* | glucose-1-phosphate cytidylyltransferase (EC:2.7.7.33) | **5.1** | **1.1** | **4.6** |
| Swit_4745 | efflux transporter, RND family, MFP subunit | **1.9** | **3.8** | **2.0** |
| Swit_4840 | Methyltransferase type 11 (EC:2.1.1.79) | **2.9** | **1.2** | **2.4** |
|  |  |  |  |  |
| **N Cell motility** | | | | |
| Swit_0065* | CheA signal transduction histidine kinase (EC:2.7.13.3) | **6.1** | **3.7** | **1.7** |
| Swit_0068* | response regulator receiver modulated CheB methylesterase (EC:3.1.1.61) | **5.9** | **3.3** | **1.8** |
| Swit_0213 | flagellar hook-associated 2 domain protein | **3.5** | **7.1** | **2.1** |
| Swit_1259 | OmpA/MotB domain protein | **3.6** | **4.5** | **1.2** |
| Swit_1260 | Flagellar motor component-like protein | **3.7** | **4.5** | **1.2** |
| Swit_1261 | flagellin domain protein | **5.5** | **4.9** | **1.1** |
| Swit_1262 | flagellar hook-associated protein FlgK | **3.3** | **4.4** | **1.3** |
| Swit_1263* | Rod binding-like protein | **3.6** | **6.9** | **1.9** |
| Swit_1264 | flagellar P-ring protein | **12.1** | **7.1** | **1.7** |
| Swit_1265 | flagellar L-ring protein | **5.6** | **10.6** | **1.9** |
| Swit_1266 | flagellar basal-body rod protein FlgG | **6.1** | **8.9** | **1.5** |
| Swit_1267 | flagellar basal-body rod protein FlgF | **6.1** | **7.3** | **1.2** |
| Swit_1268 | flagellar basal body FlaE domain protein | **9.3** | **9.2** | **1.0** |
| Swit_1269 | flagellar hook capping protein | **5.3** | **8.4** | **1.6** |
| Swit_1270 | flagellar basal-body rod protein FlgC | **9.0** | **9.8** | **1.1** |
| Swit_1271 | flagellar basal-body rod protein FlgB | **6.2** | **6.6** | **1.1** |
| Swit_1272 | MotA/TolQ/ExbB proton channel | **21.8** | **14.9** | **1.5** |
| Swit_1274* | hypothetical protein | **5.6** | **8.4** | **1.5** |
| Swit_1279* | flagellar biosynthesis protein FlhA | **14.0** | **8.8** | **1.6** |
| Swit_1283 | flagellin domain protein | **11.0** | **13.8** | **1.3** |
| Swit_1284 | flagellin domain protein | **7.0** | **10.7** | **1.5** |
| Swit_1287* | flagellar M-ring protein FliF | **9.6** | **6.4** | **1.5** |
| Swit_1288 | flagellar motor switch protein FliG | **7.0** | **4.6** | **1.5** |
| Swit_1289* | hypothetical protein | **9.9** | **15.4** | **1.6** |
| Swit_1290* | ATPase, FliI/YscN family (EC:3.6.3.14) | **6.4** | **7.4** | **1.2** |
| Swit_1293 | flagellar basal body-associated protein FliL | **5.1** | **6.1** | **1.2** |
| Swit_1313* | methyl-accepting chemotaxis sensory transducer | **7.5** | **5.3** | **1.4** |
| Swit_1458 | flagellar motor switch protein FliM | **7.9** | **3.9** | **2.0** |
| Swit_2932* | methyl-accepting chemotaxis sensory transducer | **7.4** | **1.1** | **8.0** |
| Swit_3186* | response regulator receiver modulated CheB methylesterase (EC:3.1.1.61) | **3.9** | **6.4** | **1.6** |
| Swit_3510* | type II secretion system protein | **3.4** | **3.6** | **1.0** |
| Swit_3822* | methyl-accepting chemotaxis sensory transducer | **4.0** | **3.7** | **1.1** |
| Swit_3980* | methyl-accepting chemotaxis sensory transducer | **9.8** | **7.5** | **1.3** |
| Swit_4628* | methyl-accepting chemotaxis sensory transducer | **6.8** | **5.2** | **1.3** |
| Swit_4864* | type II secretion system protein | **2.5** | **4.7** | **1.9** |
| Swit_4867 | hypothetical protein | **4.3** | **7.4** | **1.7** |
|  |  |  |  |  |
| **O Posttranslational modification, protein turnover, chaperones** | | | | |
| Swit_0145 | Glutathione S-transferase, N-terminal domain (EC:2.5.1.18) | **2.2** | **2.8** | **6.1** |
| Swit_0791 | hypothetical protein | **5.8** | **6.3** | **1.1** |
| Swit_1146 | ATP-dependent protease La (EC:3.4.21.53) | **2.7** | **2.0** | **1.3** |
| Swit_1147 | Molecular chaperone (small heat shock protein)-like protein | **4.4** | **2.6** | **1.7** |
| Swit_1249 | chaperone protein DnaJ | **1.8** | **3.1** | **1.7** |
| Swit_1250 | chaperone protein DnaK | **3.3** | **3.7** | **1.1** |
| Swit_1263* | Rod binding-like protein | **3.6** | **6.9** | **1.9** |
| Swit_1274* | hypothetical protein | **5.6** | **8.4** | **1.5** |
| Swit_2190* | peptidase S14, ClpP | **2.7** | **2.6** | **7.2** |
| Swit_2245 | Glutathione S-transferase, N-terminal domain (EC:2.5.1.18) | **3.0** | **1.2** | **3.5** |
| Swit_2918 | FeS assembly protein SufB; cysteine desulfurase activator complex subunit SufB | **2.1** | **1.4** | **3.0** |
| Swit_3375 | chaperonin Cpn10 | **2.3** | **3.8** | **1.7** |
| Swit_3376 | chaperonin GroEL | **1.1** | **2.3** | **2.4** |
| Swit_3457 | Glutathione S-transferase, N-terminal domain (EC:2.5.1.18) | **5.5** | **1.3** | **6.9** |
| Swit_3921* | glutamate-ammonia ligase adenylyltransferase | **3.6** | **2.7** | **1.3** |
| Swit_4377 | heat shock protein HslVU, ATPase subunit HslU | **2.5** | **3.1** | **1.2** |
| Swit_4712 | glutaredoxin-like protein | **3.1** | **2.9** | **1.1** |
| Swit_4870* | peptidase A24A, prepilin type IV (EC:3.4.23.43) | **4.8** | **6.6** | **1.4** |
| Swit_5306 | heat shock protein DnaJ domain protein | **7.0** | **4.7** | **1.5** |
| Swit_5351 | heat shock protein Hsp90 | **1.9** | **3.3** | **1.7** |
|  |  |  |  |  |
| **P Inorganic ion transport and metabolism** | | | | |
| Swit_0277 | TonB-dependent receptor | **1.2** | **3.2** | **3.9** |
| Swit_0535 | TonB-dependent receptor | **3.4** | **4.1** | **1.2** |
| Swit_0687 | TonB-dependent receptor | **2.1** | **7.9** | **3.7** |
| Swit_0914 | TonB-dependent receptor | **3.1** | **1.5** | **2.0** |
| Swit_1066 | TonB-dependent receptor | **19.8** | **1.2** | **17.1** |
| Swit_1067* | Vanillate monooxygenase (EC:1.14.13.82) | **38.0** | **1.5** | **25.3** |
| Swit_1901 | TonB-dependent receptor | **3.4** | **4.7** | **1.4** |
| Swit_2251* | Rieske (2Fe-2S) domain protein | **13.1** | **2.1** | **6.3** |
| Swit_2420 | TonB-dependent receptor | **7.5** | **12.2** | **1.6** |
| Swit_2477 | TonB-dependent receptor | **1.5** | **6.3** | **4.2** |
| Swit_2516 | sulfatase (EC:3.1.6.1) | **3.0** | **1.4** | **4.1** |
| Swit_2533 | major facilitator superfamily MFS_1 | **3.8** | **1.2** | **3.1** |
| Swit_2617 | copper resistance D domain protein | **4.7** | **2.9** | **1.6** |
| Swit_2634* | Rieske (2Fe-2S) domain protein (EC:1.14.12.10) | **1.7** | **19.0** | **10.9** |
| Swit_2933 | Superoxide dismutase (EC:1.15.1.1) | **1.6** | **3.0** | **1.9** |
| Swit_3043 | sulfatase (EC:3.1.6.1) | **1.9** | **4.3** | **2.2** |
| Swit_3044 | TonB-dependent receptor | **2.7** | **5.1** | **1.9** |
| Swit_3048 | TonB-dependent receptor | **1.5** | **3.0** | **2.0** |
| Swit_3164 | Superoxide dismutase (EC:1.15.1.1) | **7.9** | **6.9** | **1.1** |
| Swit_3263 | TonB-dependent receptor | **20.7** | **1.0** | **19.9** |
| Swit_3264* | Vanillate monooxygenase (EC:1.14.13.82) | **16.7** | **1.0** | **17.0** |
| Swit_3266* | Rieske (2Fe-2S) domain protein (EC:1.14.13.82) | **8.6** | **1.1** | **9.3** |
| Swit_3560 | TonB-dependent siderophore receptor | **1.9** | **4.2** | **2.2** |
| Swit_3723 | Rhodanese domain protein | **1.5** | **5.1** | **3.3** |
| Swit_3918 | TonB-dependent receptor | **1.3** | **2.2** | **3.0** |
| Swit_4025 | TonB-dependent siderophore receptor | **1.4** | **4.2** | **2.9** |
| Swit_4088 | TonB-dependent receptor | **3.1** | **1.1** | **3.0** |
| Swit_4197 | TonB-dependent receptor | **1.3** | **2.5** | **3.2** |
| Swit_4368 |  | **7.6** | **4.0** | **1.9** |
| Swit_4696 | TonB-dependent receptor | **1.2** | **2.3** | **2.8** |
| Swit_4753 | cation diffusion facilitator family transporter | **3.4** | **2.8** | **1.2** |
| Swit_4781 | TonB-dependent receptor | **1.2** | **4.3** | **3.6** |
| Swit_4852 | TonB-dependent receptor | **1.8** | **5.3** | **3.0** |
| Swit_5394 | Na+/H+ antiporter | **1.0** | **2.5** | **2.5** |
|  |  |  |  |  |
| **Q Secondary metabolites biosynthesis, transport and catabolism** | | | | |
| Swit_0088* | acyl carrier protein | **3.5** | **3.6** | **1.0** |
| Swit_0380* | short-chain dehydrogenase/reductase SDR (EC:1.1.1.69) | **2.1** | **7.4** | **3.6** |
| Swit_0769* | short-chain dehydrogenase/reductase SDR | **1.8** | **6.4** | **3.6** |
| Swit_0976 | Muconolactone Delta-isomerase (EC:5.3.3.4) | **13.6** | **1.1** | **12.3** |
| Swit_0977 | catechol 1,2-dioxygenase (EC:1.13.11.1) | **9.8** | **2.1** | **20.3** |
| Swit_1557 | Homogentisate 1,2-dioxygenase (EC:1.13.11.5) | **2.9** | **2.2** | **6.4** |
| Swit_2111 | 4-oxalocrotonate decarboxylase (EC:4.2.1.80) | **8.1** | **16.6** | **2.0** |
| Swit_2112 | Acetaldehyde dehydrogenase-like protein (EC:1.2.1.10) | **4.1** | **9.1** | **2.2** |
| Swit_2416 | amidohydrolase | **3.2** | **5.3** | **1.6** |
| Swit_2635 | 2-chlorobenzoate 1,2-dioxygenase (EC:1.14.12.10,EC:1.14.12.13) | **4.4** | **23.2** | **5.3** |
| Swit_2636* | short-chain dehydrogenase/reductase SDR (EC:1.3.1.25) | **2.3** | **5.5** | **2.3** |
| Swit_3047 | Phytanoyl-CoA dioxygenase | **1.6** | **3.0** | **1.9** |
| Swit_3057 | aromatic-ring-hydroxylating dioxygenase, beta subunit (EC:1.14.12.19) | **1.8** | **3.3** | **1.8** |
| Swit_3915* | histone deacetylase superfamily | **2.6** | **6.1** | **2.3** |
| Swit_4335* | short-chain dehydrogenase/reductase SDR (EC:1.1.1.100) | **13.1** | **1.3** | **17.4** |
| Swit_4924 | Acetaldehyde dehydrogenase-like protein (EC:1.2.1.10) | **10.3** | **18.5** | **1.8** |
| Swit_4925 | 4-oxalocrotonate decarboxylase (EC:4.2.1.80) | **21.3** | **38.1** | **1.8** |
| Swit_5102 | Cupin 2, conserved barrel domain protein (EC:1.13.11.4) (gentisate 1,2-dioxygenase Coronado 2012) | **1.1** | **25.9** | **29.7** |
| Swit_5291* | short-chain dehydrogenase/reductase SDR | **4.5** | **2.5** | **1.8** |
|  |  |  |  |  |
| **R General function prediction only** | | | | |
| Swit_0313 | amidohydrolase 2 | **3.1** | **1.1** | **3.5** |
| Swit_0380* | short-chain dehydrogenase/reductase SDR (EC:1.1.1.69) | **2.1** | **7.4** | **3.6** |
| Swit_0686 | beta-lactamase domain protein | **1.9** | **4.0** | **2.2** |
| Swit_0769* | short-chain dehydrogenase/reductase SDR | **1.8** | **6.4** | **3.6** |
| Swit_0793 | Acetyltransferase (isoleucine patch superfamily)-like protein (EC:2.3.1.18) | **6.0** | **5.7** | **1.0** |
| Swit_0873 | PEBP family protein | **3.0** | **4.5** | **1.5** |
| Swit_0975* | muconate and chloromuconate cycloisomerase (EC:5.5.1.1) | **27.5** | **2.0** | **13.8** |
| Swit_0978 | 3-oxoadipate enol-lactonase (EC:3.1.1.24) | **8.4** | **2.1** | **17.7** |
| Swit_1067* | Vanillate monooxygenase (EC:1.14.13.82) | **38.0** | **1.5** | **25.3** |
| Swit_1680 | Glyoxalase/bleomycin resistance protein/dioxygenase | **12.3** | **3.3** | **3.7** |
| Swit_1898 | SSS sodium solute transporter superfamily | **4.0** | **1.7** | **2.3** |
| Swit_2183 | plasmid maintenance system killer | **2.9** | **1.3** | **3.7** |
| Swit_2191 | phage portal protein, lambda family | **3.3** | **1.1** | **3.7** |
| Swit_2193 | phage terminase GpA | **3.4** | **1.5** | **5.1** |
| Swit_2251* | Rieske (2Fe-2S) domain protein | **13.1** | **2.1** | **6.3** |
| Swit_2616 | copper resistance protein CopC | **4.2** | **4.6** | **1.1** |
| Swit_2634* | Rieske (2Fe-2S) domain protein (EC:1.14.12.10) | **1.7** | **19.0** | **10.9** |
| Swit_2636* | short-chain dehydrogenase/reductase SDR (EC:1.3.1.25) | **2.3** | **5.5** | **2.3** |
| Swit_2652 | polysaccharide biosynthesis protein | **1.6** | **3.0** | **1.9** |
| Swit_2654 | hypothetical protein | **1.7** | **3.2** | **1.9** |
| Swit_2810 | flavoprotein WrbA; Trp binding | **17.2** | **2.8** | **6.1** |
| Swit_2811 | Pirin domain protein | **5.3** | **1.2** | **4.3** |
| Swit_3018 | Alcohol dehydrogenase GroES domain protein (EC:1.1.1.1) | **1.4** | **1.9** | **2.7** |
| Swit_3019 | Glyoxalase/bleomycin resistance protein/dioxygenase | **2.2** | **5.8** | **2.6** |
| Swit_3183 | oxidoreductase domain protein | **4.5** | **1.7** | **2.7** |
| Swit_3199 | conserved hypothetical protein 730 | **1.0** | **3.0** | **3.1** |
| Swit_3264* | Vanillate monooxygenase (EC:1.14.13.82) | **16.7** | **1.0** | **17.0** |
| Swit_3266* | Rieske (2Fe-2S) domain protein (EC:1.14.13.82) | **8.6** | **1.1** | **9.3** |
| Swit_3903* | diacylglycerol kinase, catalytic region | **1.7** | **3.2** | **1.9** |
| Swit_4201* | D-isomer specific 2-hydroxyacid dehydrogenase, NAD-binding | **6.5** | **1.9** | **3.4** |
| Swit_4323* | D-isomer specific 2-hydroxyacid dehydrogenase, NAD-binding | **3.8** | **1.8** | **2.1** |
| Swit_4335* | short-chain dehydrogenase/reductase SDR (EC:1.1.1.100) | **13.1** | **1.3** | **17.4** |
| Swit_4396 | GCN5-related N-acetyltransferase | **1.6** | **3.5** | **5.8** |
| Swit_4433 | Haloacid dehalogenase domain protein hydrolase | **4.0** | **4.4** | **1.1** |
| Swit_4436 | Bacteriophage terminase large (ATPase) subunit and inactivated derivatives-like protein | **2.0** | **15.3** | **30.5** |
| Swit_4504 | 2-nitropropane dioxygenase, NPD (EC:1.3.1.-) | **1.4** | **2.1** | **2.9** |
| Swit_4543 | glycosyl transferase, family 2 | **2.3** | **1.3** | **2.9** |
| Swit_4571 | acetyltransferase-like protein | **5.3** | **3.8** | **1.4** |
| Swit_4842 | amine oxidase | **4.1** | **1.4** | **2.8** |
| Swit_4902 | Glyoxalase/bleomycin resistance protein/dioxygenase (EC:1.13.11.39), dbfB | **2.8** | **7.6** | **2.7** |
| Swit_5087 | Pseudogene | **3.0** | **1.0** | **3.1** |
| Swit_5288* | PEBP family protein | **2.3** | **1.3** | **2.8** |
| Swit_5291* | short-chain dehydrogenase/reductase SDR | **4.5** | **2.5** | **1.8** |
| Swit_5345* | Alcohol dehydrogenase GroES domain protein (EC:1.1.1.284) | **3.2** | **1.2** | **2.7** |
| Swit_5385 | protein of unknown function UPF0005 | **8.6** | **2.5** | **3.4** |
|  |  |  |  |  |
| **S Function unknown** | | | | |
| Swit_0155 | protein of unknown function DUF1044 | **3.2** | **1.5** | **2.1** |
| Swit_0490 | hypothetical protein | **1.1** | **2.7** | **2.9** |
| Swit_0556 | protein of unknown function DUF1178 | **3.4** | **1.6** | **2.2** |
| Swit_0634 | protein of unknown function DUF465 | **4.4** | **2.4** | **1.8** |
| Swit_0690 | 40-residue YVTN family beta-propeller repeat protein | **1.2** | **7.0** | **5.7** |
| Swit_0702 | protein of unknown function DUF779 | **1.4** | **5.3** | **3.9** |
| Swit_0785 | protein of unknown function DUF323 | **3.2** | **1.3** | **4.3** |
| Swit_0862 | phasin family protein | **6.6** | **1.7** | **3.8** |
| Swit_0878 | protein of unknown function DUF1452 | **4.8** | **1.2** | **4.0** |
| Swit_0880 | hypothetical protein | **4.0** | **1.2** | **3.2** |
| Swit_1093 | hypothetical protein (**Zeta toxin †**) | **3.4** | **3.6** | **1.1** |
| Swit_1108 | Activator of Hsp90 ATPase 1 family protein | **1.9** | **1.5** | **2.9** |
| Swit_1218 | TPR repeat-containing protein | **1.8** | **2.0** | **3.5** |
| Swit_1361 | hypothetical protein | **2.6** | **1.1** | **2.8** |
| Swit_1451 | protein of unknown function DUF156 | **1.2** | **2.9** | **2.5** |
| Swit_1813 | hypothetical protein | **4.3** | **20.9** | **4.8** |
| Swit_2189 | hypothetical protein | **3.6** | **2.5** | **9.1** |
| Swit_2265 | Antibiotic biosynthesis monooxygenase; autoinducer-2 degrading protein | **108.3** | **18.7** | **5.8** |
| Swit_2679 | protein of unknown function DUF1508 | **3.1** | **1.8** | **1.7** |
| Swit_2867 | Extradiol ring-cleavage dioxygenase, class III enzyme, subunit B | **3.4** | **2.6** | **1.3** |
| Swit_3430 | hypothetical protein | **5.4** | **1.6** | **3.4** |
| Swit_3563 | Uncharacterized iron-regulated membrane-like protein | **1.1** | **3.9** | **4.4** |
| Swit_3794 | hypothetical protein | **3.2** | **1.4** | **2.3** |
| Swit_3914 | protein of unknown function DUF455 | **3.6** | **1.7** | **2.2** |
| Swit_3927 | Entericidin EcnAB | **7.0** | **1.2** | **5.7** |
| Swit_3969 | protein of unknown function DUF559 | **4.3** | **1.3** | **5.7** |
| Swit_3981 | Ku family containing protein; non-homologous end-joining DNA repair | **3.6** | **1.0** | **3.7** |
| Swit_4209 | glutathione-dependent formaldehyde-activating, GFA | **4.7** | **2.4** | **11.2** |
| Swit_4375 | protein of unknown function DUF486 | **1.5** | **1.7** | **2.7** |
| Swit_4400 | protein of unknown function DUF692 | **5.7** | **1.5** | **3.8** |
| Swit_4402 | protein of unknown function DUF808 | **3.0** | **1.1** | **3.2** |
| Swit_4470 | protein of unknown function DUF526 | **6.5** | **2.8** | **2.3** |
| Swit_4471 | Protein of unknown function DUF1790 | **4.5** | **4.5** | **1.0** |
| Swit_4521 | membrane-like protein | **3.2** | **1.5** | **2.2** |
| Swit_4545 | phosphoribosyltransferase | **3.3** | **3.2** | **1.0** |
| Swit_4741 | Peptidoglycan-binding LysM | **1.2** | **2.2** | **2.6** |
| Swit_4793 | protein of unknown function DUF470 | **5.9** | **14.0** | **2.4** |
| Swit_4794 | hypothetical protein | **7.1** | **7.2** | **1.0** |
| Swit_4841 | protein of unknown function DUF1295 | **2.8** | **1.3** | **2.2** |
| Swit_4921 | protein of unknown function DUF849 | **2.4** | **3.7** | **1.5** |
| Swit_4943 | hypothetical protein | **3.0** | **1.0** | **3.1** |
| Swit_5344 | cyclase/dehydrase | **4.5** | **1.4** | **3.2** |
| Swit_5348 | conserved hypothetical protein; K08995 putative membrane protein | **3.0** | **1.4** | **2.1** |
| Swit_5386 | hypothetical protein (**membrane protein †**) | **4.7** | **1.4** | **3.3** |
|  |  |  |  |  |
| **T Signal transduction mechanisms** | | | | |
| Swit_0065* | CheA signal transduction histidine kinase (EC:2.7.13.3) | **6.1** | **3.7** | **1.7** |
| Swit_0068* | response regulator receiver modulated CheB methylesterase (EC:3.1.1.61) | **5.9** | **3.3** | **1.8** |
| Swit_0108 | diguanylate cyclase/phosphodiesterase | **4.5** | **6.2** | **1.4** |
| Swit_0175* | response regulator receiver protein | **2.3** | **1.7** | **3.8** |
| Swit_0267 | multi-sensor signal transduction histidine kinase | **1.5** | **3.3** | **2.2** |
| Swit_0404 | diguanylate cyclase | **6.4** | **6.5** | **1.0** |
| Swit_0692* | extracellular solute-binding protein, family 3 | **1.9** | **6.3** | **3.4** |
| Swit_1185 | two component, sigma54 specific, transcriptional regulator, Fis family | **5.7** | **7.2** | **1.3** |
| Swit_1285* | sigma54 specific transcriptional regulator, Fis family | **6.4** | **5.9** | **1.1** |
| Swit_1313* | methyl-accepting chemotaxis sensory transducer | **7.5** | **5.3** | **1.4** |
| Swit_1387 | integral membrane sensor signal transduction histidine kinase | **3.3** | **1.7** | **2.0** |
| Swit_1945 | putative transcriptional regulator, Crp/Fnr family | **1.6** | **12.2** | **7.7** |
| Swit_2540 | response regulator receiver protein | **2.9** | **2.6** | **7.4** |
| Swit_2596 | diguanylate cyclase | **4.0** | **3.9** | **1.0** |
| Swit_2927 | diguanylate cyclase/phosphodiesterase | **4.8** | **4.7** | **1.0** |
| Swit_2932* | methyl-accepting chemotaxis sensory transducer | **7.4** | **1.1** | **8.0** |
| Swit_3186* | response regulator receiver modulated CheB methylesterase (EC:3.1.1.61) | **3.9** | **6.4** | **1.6** |
| Swit_3187* | response regulator receiver protein | **4.2** | **6.0** | **1.4** |
| Swit_3188 | response regulator receiver protein | **6.0** | **1.2** | **5.0** |
| Swit_3381 | HPr kinase | **1.8** | **1.8** | **3.2** |
| Swit_3822* | methyl-accepting chemotaxis sensory transducer | **4.0** | **3.7** | **1.1** |
| Swit_3921* | glutamate-ammonia ligase adenylyltransferase | **3.6** | **2.7** | **1.3** |
| Swit_3980* | methyl-accepting chemotaxis sensory transducer | **9.8** | **7.5** | **1.3** |
| Swit_4432 | PAS/PAC sensor hybrid histidine kinase | **3.0** | **1.0** | **3.1** |
| Swit_4549 | HPr kinase | **1.8** | **3.5** | **6.4** |
| Swit_4627 | response regulator receiver protein | **1.7** | **2.6** | **4.3** |
| Swit_4628* | methyl-accepting chemotaxis sensory transducer | **6.8** | **5.2** | **1.3** |
| Swit_5396 | response regulator receiver protein | **6.4** | **2.2** | **2.9** |
|  |  |  |  |  |
| **U Intracellular trafficking, secretion, and vesicular transport** | | | | |
| Swit_0604 | Sporulation domain protein | **6.6** | **10.5** | **1.6** |
| Swit_1154* | RND efflux system, outer membrane lipoprotein, NodT family | **50.0** | **1.8** | **27.8** |
| Swit_1279* | flagellar biosynthesis protein FlhA | **14.0** | **8.8** | **1.6** |
| Swit_1287* | flagellar M-ring protein FliF | **9.6** | **6.4** | **1.5** |
| Swit_1289* | hypothetical protein | **9.9** | **15.4** | **1.6** |
| Swit_1290* | ATPase, FliI/YscN family (EC:3.6.3.14) | **6.4** | **7.4** | **1.2** |
| Swit_1951* | RND efflux system, outer membrane lipoprotein, NodT family | **1.1** | **5.5** | **5.0** |
| Swit_2190* | peptidase S14, ClpP | **2.7** | **2.6** | **7.2** |
| Swit_3190 | Polypeptide-transport-associated domain protein, ShlB-type | **3.0** | **1.8** | **1.7** |
| Swit_3510* | type II secretion system protein | **3.4** | **3.6** | **1.0** |
| Swit_3513 | response regulator receiver protein | **2.7** | **1.5** | **1.8** |
| Swit_3515 | Flp pilus assembly protein CpaB-like protein | **3.7** | **1.7** | **2.2** |
| Swit_4800 | virulence factor family protein | **4.6** | **10.9** | **2.4** |
| Swit_4864* | type II secretion system protein | **2.5** | **4.7** | **1.9** |
| Swit_4865 | type II secretion system protein | **9.4** | **7.9** | **1.2** |
| Swit_4866 | response regulator receiver protein | **5.2** | **5.8** | **1.1** |
| Swit_4868 | type II and III secretion system protein | **6.8** | **10.0** | **1.5** |
| Swit_4869 | SAF domain | **9.1** | **4.5** | **2.0** |
| Swit_4870* | peptidase A24A, prepilin type IV (EC:3.4.23.43) | **4.8** | **6.6** | **1.4** |
| Swit_5004 | VirB8 family protein | **1.4** | **3.0** | **4.0** |
| Swit_5160 | TraD | **1.8** | **4.9** | **2.7** |
| Swit_5371 | Type-IV secretion system protein TraC | **5.6** | **1.0** | **5.5** |
|  |  |  |  |  |
| **V Defense mechanisms** | | | | |
| Swit_1153 | transporter, hydrophobe/amphiphile efflux-1 (HAE1) family | **53.2** | **2.9** | **18.1** |
| Swit_1952 | transporter, hydrophobe/amphiphile efflux-1 (HAE1) family | **1.1** | **5.3** | **5.0** |
| Swit_2345 | ABC transporter related (EC:3.6.3.-) | **1.8** | **1.5** | **2.8** |
| Swit_4053 | secretion protein HlyD family protein | **2.0** | **1.5** | **3.1** |
| Swit_4464 | N-acetylmuramoyl-L-alanine amidase, family 2 (EC:3.5.1.28) | **5.0** | **1.3** | **6.7** |
| Swit_4845 | ABC transporter related | **1.5** | **3.3** | **2.2** |
|  |  |  |  |  |
| **COG unassigned** | | | | |
| Swit_0064 | hypothetical protein | **1.4** | **3.6** | **4.9** |
| Swit_0071 | hypothetical protein (**plasmid stabilization protein †**) | **7.1** | **1.2** | **6.0** |
| Swit_0072 | hypothetical protein | **7.5** | **1.5** | **5.0** |
| Swit_0147 | hypothetical protein | **2.4** | **2.1** | **5.0** |
| Swit_0202 | hypothetical protein | **1.7** | **1.7** | **2.9** |
| Swit_0230 | hypothetical protein | **3.3** | **1.9** | **1.7** |
| Swit_0239 | hypothetical protein | **6.7** | **1.1** | **5.9** |
| Swit_0255 | hypothetical protein | **1.8** | **1.5** | **2.7** |
| Swit_0266 | UspA domain protein | **1.8** | **10.1** | **5.5** |
| Swit_0428 | hypothetical protein (**nickel uptake transporter family protein, partial †**) | **1.5** | **1.9** | **2.8** |
| Swit_0524 | hypothetical protein | **3.5** | **1.7** | **2.1** |
| Swit_0545 | hypothetical protein (**general stress protein CsbD †**) | **7.0** | **1.0** | **6.8** |
| Swit_0565 | type IV pilus assembly PilZ | **3.1** | **4.1** | **1.3** |
| Swit_0655 | hypothetical protein | **5.9** | **1.4** | **4.4** |
| Swit_0656 | hypothetical protein | **6.3** | **1.2** | **5.2** |
| Swit_0689 | hypothetical protein | **1.3** | **10.1** | **7.8** |
| Swit_0798 | peptidase C14, caspase catalytic subunit p20 | **1.3** | **4.5** | **5.9** |
| Swit_0799 | hypothetical protein | **1.1** | **3.8** | **3.4** |
| Swit_0800 | hypothetical protein | **1.5** | **2.8** | **4.2** |
| Swit_0858 | Hemerythrin HHE cation binding domain protein | **5.2** | **1.4** | **3.8** |
| Swit_0867 | hypothetical protein | **4.8** | **1.7** | **2.9** |
| Swit_0869 | hypothetical protein | **3.0** | **3.8** | **1.3** |
| Swit_0954 | hypothetical protein | **3.0** | **1.2** | **3.8** |
| Swit_0995 | PRC-barrel domain protein | **5.4** | **1.1** | **4.7** |
| Swit_1107 | hypothetical protein | **36.7** | **3.5** | **10.6** |
| Swit_1242 | hypothetical protein | **3.2** | **2.7** | **1.2** |
| Swit_1258 | hypothetical protein | **4.3** | **3.5** | **1.2** |
| Swit_1273 | hypothetical protein | **11.4** | **30.4** | **2.7** |
| Swit_1275 | putative anti-sigma-28 factor, FlgM | **3.5** | **8.0** | **2.3** |
| Swit_1276 | hypothetical protein | **5.4** | **14.1** | **2.6** |
| Swit_1282 | hypothetical protein | **6.3** | **13.4** | **2.1** |
| Swit_1291 | hypothetical protein | **17.0** | **6.6** | **2.6** |
| Swit_1292 | flagellar hook-length control protein | **5.0** | **11.5** | **2.3** |
| Swit_1294 | hypothetical protein | **7.2** | **1.7** | **4.1** |
| Swit_1295 | hypothetical protein | **3.2** | **2.0** | **1.6** |
| Swit_1418 | hypothetical protein | **2.5** | **4.0** | **1.6** |
| Swit_1507 | 17 kDa surface antigen | **3.4** | **2.0** | **1.7** |
| Swit_1515 | hypothetical protein | **4.4** | **4.9** | **1.1** |
| Swit_1805 | hypothetical protein | **3.5** | **7.2** | **2.0** |
| Swit_1806 | hypothetical protein | **4.5** | **6.5** | **1.4** |
| Swit_1812 | hypothetical protein | **3.0** | **9.9** | **3.3** |
| Swit_1934 | hypothetical protein | **4.7** | **5.1** | **1.1** |
| Swit_1946 | hypothetical protein | **1.1** | **9.0** | **8.0** |
| Swit_2170 | hypothetical protein | **3.0** | **4.1** | **12.3** |
| Swit_2171 | Peptidase M15A | **2.0** | **3.1** | **6.2** |
| Swit_2172 | hypothetical protein | **2.5** | **2.1** | **5.2** |
| Swit_2174 | hypothetical protein | **2.1** | **2.2** | **4.7** |
| Swit_2175 | hypothetical protein | **1.1** | **10.3** | **10.9** |
| Swit_2176 | hypothetical protein | **1.5** | **3.1** | **4.6** |
| Swit_2177 | hypothetical protein | **2.4** | **4.3** | **10.3** |
| Swit_2178 | hypothetical protein | **1.7** | **3.0** | **5.3** |
| Swit_2179 | hypothetical protein | **2.4** | **1.9** | **4.5** |
| Swit_2180 | hypothetical protein | **3.4** | **2.1** | **1.6** |
| Swit_2181 | hypothetical protein | **2.0** | **3.1** | **6.2** |
| Swit_2182 | hypothetical protein | **1.9** | **2.3** | **4.3** |
| Swit_2185 | hypothetical protein | **2.2** | **2.3** | **5.2** |
| Swit_2186 | hypothetical protein | **3.2** | **3.4** | **11.2** |
| Swit_2187 | hypothetical protein | **1.4** | **3.1** | **4.3** |
| Swit_2188 | hypothetical protein | **4.4** | **1.9** | **8.6** |
| Swit_2194 | hypothetical protein | **4.3** | **1.0** | **4.2** |
| Swit_2207 | hypothetical protein | **1.8** | **2.2** | **3.8** |
| Swit_2211 | hypothetical protein | **2.1** | **50.6** | **24.5** |
| Swit_2212 | hypothetical protein | **3.1** | **3.0** | **9.3** |
| Swit_2213 | hypothetical protein | **1.8** | **14.8** | **8.1** |
| Swit_2324 | protein of unknown function UPF0057 | **3.3** | **1.1** | **3.1** |
| Swit_2325 | hypothetical protein | **4.0** | **1.0** | **3.8** |
| Swit_2334 | protein of unknown function DUF1328 | **10.5** | **1.7** | **6.2** |
| Swit_2344 | hypothetical protein | **4.3** | **5.2** | **1.2** |
| Swit_2398 | hypothetical protein | **2.2** | **1.6** | **3.5** |
| Swit_2423 | hypothetical protein | **3.4** | **3.5** | **1.0** |
| Swit_2527 | hypothetical protein | **4.4** | **2.7** | **1.6** |
| Swit_2639 | hypothetical protein | **2.8** | **3.4** | **1.2** |
| Swit_2646 |  | **2.9** | **5.0** | **1.7** |
| Swit_2647 | hypothetical protein | **4.0** | **6.1** | **1.5** |
| Swit_2651 | hypothetical protein | **2.9** | **6.1** | **2.1** |
| Swit_2730 | hypothetical protein | **2.6** | **1.3** | **3.4** |
| Swit_2787 | Sel1 domain protein repeat-containing protein | **4.0** | **2.7** | **1.5** |
| Swit_2809 | hypothetical protein | **4.1** | **1.3** | **3.3** |
| Swit_2863 | hypothetical protein | **3.8** | **2.4** | **1.6** |
| Swit_2942 | hypothetical protein | **1.6** | **2.6** | **4.1** |
| Swit_2968 | Calcium-binding EF-hand-containing protein | **1.7** | **2.1** | **3.4** |
| Swit_3008 | hypothetical protein | **1.1** | **51.5** | **48.5** |
| Swit_3117 | hypothetical protein | **23.7** | **429.9** | **18.2** |
| Swit_3138 | hypothetical protein | **4.0** | **5.2** | **1.3** |
| Swit_3193 | hypothetical protein | **5.1** | **1.3** | **6.6** |
| Swit_3223 | hypothetical protein (**glyoxalase/bleomycin resistance protein/dioxygenase †**) | **5.5** | **3.0** | **1.9** |
| Swit_3377 | hypothetical protein | **1.1** | **2.4** | **2.8** |
| Swit_3596 | hypothetical protein | **2.9** | **1.5** | **2.0** |
| Swit_3680 | hypothetical protein | **1.1** | **4.7** | **5.2** |
| Swit_3718 | hypothetical protein | **2.3** | **1.8** | **4.2** |
| Swit_3777 | hypothetical protein | **10.2** | **6.3** | **1.6** |
| Swit_3778 | hypothetical protein | **1.0** | **3.0** | **3.0** |
| Swit_3923 | hypothetical protein | **13.1** | **2.5** | **5.2** |
| Swit_3971 | hypothetical protein | **3.4** | **2.3** | **7.9** |
| Swit_3984 | hypothetical protein | **2.9** | **2.2** | **1.3** |
| Swit_4005 | sulfotransferase | **1.1** | **2.7** | **2.9** |
| Swit_4069 | hypothetical protein | **4.8** | **4.0** | **1.2** |
| Swit_4096 | Hemerythrin HHE cation binding domain protein | **5.0** | **1.2** | **4.0** |
| Swit_4237 | anti-ECFsigma factor, ChrR | **2.6** | **2.9** | **7.4** |
| Swit_4284 | malonate/sodium symporter MadM subunit | **3.7** | **2.4** | **1.5** |
| Swit_4285 | malonate transporter MadL subunit | **10.9** | **2.3** | **4.8** |
| Swit_4286 | hypothetical protein (**malonate decarboxylase subunit alpha** **†**) | **7.7** | **2.3** | **3.3** |
| Swit_4287 | malonate decarboxylase delta subunit | **6.3** | **2.3** | **2.8** |
| Swit_4289 | malonate decarboxylase gamma subunit | **6.0** | **1.5** | **4.0** |
| Swit_4290 | hypothetical protein | **8.9** | **1.8** | **16.0** |
| Swit_4363 | methylamine dehydrogenase heavy subunit (EC:1.4.99.3) | **35.5** | **15.5** | **2.3** |
| Swit_4364 | methylamine dehydrogenase accessory protein MauD | **15.5** | **9.4** | **1.6** |
| Swit_4365 | Amine dehydrogenase (EC:1.4.99.3) | **12.8** | **7.4** | **1.7** |
| Swit_4366 | cytochrome c, class I | **10.3** | **5.8** | **1.8** |
| Swit_4367 | hypothetical protein (**cytochrome C †**) | **15.8** | **3.3** | **4.8** |
| Swit_4389 | hypothetical protein | **6.2** | **7.7** | **1.2** |
| Swit_4397 | hypothetical protein (**HNH endonuclease †**) | **2.8** | **18.6** | **6.7** |
| Swit_4399 | hypothetical protein | **3.9** | **2.2** | **1.8** |
| Swit_4401 | hypothetical protein | **3.1** | **1.5** | **2.0** |
| Swit_4412 | type IV pilus assembly PilZ | **6.0** | **8.1** | **1.4** |
| Swit_4423 | hypothetical protein | **4.8** | **5.7** | **1.2** |
| Swit_4434 | hypothetical protein | **1.1** | **8.4** | **7.7** |
| Swit_4437 | hypothetical protein | **3.0** | **2.7** | **8.1** |
| Swit_4440 | hypothetical protein (**DNA packaging protein Gp2** **†**) | **1.6** | **2.7** | **4.4** |
| Swit_4441 | hypothetical protein | **2.3** | **1.9** | **4.3** |
| Swit_4443 | hypothetical protein | **1.1** | **3.5** | **3.9** |
| Swit_4444 | hypothetical protein | **3.0** | **1.8** | **5.3** |
| Swit_4446 | hypothetical protein | **1.6** | **3.8** | **6.0** |
| Swit_4447 | hypothetical protein | **2.8** | **1.5** | **4.3** |
| Swit_4450 | hypothetical protein | **2.4** | **1.4** | **3.3** |
| Swit_4451 | hypothetical protein | **1.6** | **6.5** | **10.5** |
| Swit_4452 | hypothetical protein | **1.8** | **3.8** | **6.8** |
| Swit_4455 | hypothetical protein | **11.7** | **3.6** | **3.3** |
| Swit_4456 | hypothetical protein | **1.9** | **2.1** | **4.0** |
| Swit_4460 | hypothetical protein | **4.0** | **1.0** | **4.0** |
| Swit_4461 | hypothetical protein | **1.5** | **5.6** | **8.5** |
| Swit_4462 | hypothetical protein | **2.3** | **4.1** | **9.4** |
| Swit_4463 | hypothetical protein | **3.4** | **5.8** | **19.7** |
| Swit_4466 | hypothetical protein | **3.0** | **1.3** | **4.0** |
| Swit_4475 | hypothetical protein | **7.8** | **1.3** | **5.8** |
| Swit_4478 | hypothetical protein | **5.4** | **3.8** | **1.4** |
| Swit_4485 | H+-transporting two-sector ATPase, B/B' subunit (EC:3.6.3.14) | **1.7** | **1.8** | **3.1** |
| Swit_4487 | hypothetical protein | **1.8** | **4.1** | **2.2** |
| Swit_4490 | hypothetical protein | **1.2** | **35.4** | **28.5** |
| Swit_4497 | hypothetical protein | **10.0** | **1.4** | **14.3** |
| Swit_4546 | hypothetical protein | **26.2** | **2.9** | **76.8** |
| Swit_4547 | hypothetical protein | **5.1** | **1.8** | **9.1** |
| Swit_4564 | hypothetical protein | **7.0** | **1.0** | **6.8** |
| Swit_4591 | hypothetical protein | **2.2** | **1.4** | **3.0** |
| Swit_4605 | hypothetical protein | **12.3** | **9.5** | **1.3** |
| Swit_4626 | transcriptional regulator, TetR family | **1.8** | **2.6** | **4.7** |
| Swit_4646 | hypothetical protein | **3.4** | **1.3** | **4.3** |
| Swit_4647 | hypothetical protein | **12.5** | **3.9** | **3.2** |
| Swit_4678 | hypothetical protein | **2.3** | **3.3** | **7.5** |
| Swit_4749 | Transglycosylase-associated protein | **7.9** | **1.8** | **4.5** |
| Swit_4750 | protein of unknown function DUF1153 | **4.9** | **6.5** | **1.3** |
| Swit_4761 | hypothetical protein | **5.4** | **4.9** | **1.1** |
| Swit_4763 | hypothetical protein | **3.9** | **1.1** | **3.6** |
| Swit_4779 | hypothetical protein | **1.2** | **3.4** | **2.9** |
| Swit_4795 | hypothetical protein | **8.9** | **11.1** | **1.2** |
| Swit_4802 | hypothetical protein | **20.9** | **36.5** | **1.7** |
| Swit_4826 | hypothetical protein | **6.2** | **9.0** | **1.4** |
| Swit_4903 | transposase IS3/IS911 family protein | **3.8** | **7.1** | **1.9** |
| Swit_4904 | hypothetical protein (**Fis family transcriptional regulator †**) | **2.9** | **7.7** | **2.7** |
| Swit_4912 | hypothetical protein | **1.1** | **28.7** | **31.7** |
| Swit_4942 | hypothetical protein | **4.1** | **1.2** | **4.7** |
| Swit_5069 | hypothetical protein | **5.1** | **3.8** | **1.3** |
| Swit_5084 | hypothetical protein | **3.2** | **2.3** | **1.4** |
| Swit_5105 | hypothetical protein (**integrase catalytic subunit, partial †**) | **1.8** | **6.0** | **3.3** |
| Swit_5110 | hypothetical protein (**integrase, partial †**) | **2.2** | **5.0** | **2.2** |
| Swit_5113 | hypothetical protein | **1.4** | **2.0** | **2.8** |
| Swit_5161 | hypothetical protein | **3.0** | **2.5** | **7.4** |
| Swit_5166 | hypothetical protein | **1.7** | **11.8** | **7.0** |
| Swit_5189 | hypothetical protein | **1.1** | **6.1** | **5.8** |
| Swit_5192 | hypothetical protein | **1.8** | **3.7** | **2.0** |
| Swit_5193 | hypothetical protein (**PRTRC system protein B †**) | **8.2** | **12.0** | **1.5** |
| Swit_5194 | hypothetical protein | **4.9** | **19.8** | **4.1** |
| Swit_5206 | hypothetical protein | **2.6** | **10.4** | **3.9** |
| Swit_5225 | hypothetical protein | **7.4** | **1.4** | **5.5** |
| Swit_5226 | hypothetical protein | **13.9** | **2.4** | **5.9** |
| Swit_5255 | hypothetical protein | **1.2** | **2.8** | **3.4** |
| Swit_5263 | hypothetical protein | **7.5** | **5.1** | **1.5** |
| Swit_5295 | Alternative oxidase | **1.3** | **4.9** | **6.2** |
| Swit_5310 | hypothetical protein | **2.9** | **1.9** | **5.7** |
| Swit_5331 | hypothetical protein | **1.8** | **2.0** | **3.5** |
| Swit_5347 | hypothetical protein | **1.4** | **2.7** | **3.9** |
| Swit_5350 | protein of unknown function DUF465 | **4.1** | **1.2** | **5.1** |
| Swit_5360 | hypothetical protein | **2.0** | **3.9** | **1.9** |
| Swit_5364 | hypothetical protein | **36.5** | **3.4** | **10.8** |
| Swit_5365 | Type IV conjugative transfer system protein TraL | **188.6** | **7.1** | **26.4** |
| Swit_5366 | TraE family protein | **149.9** | **27.4** | **5.5** |
| Swit_5367 | hypothetical protein (**conjugal transfer protein TraK †**) | **15.3** | **3.4** | **4.5** |
| Swit_5368 | TraB pilus assembly family protein | **11.7** | **1.5** | **8.0** |
| Swit_5369 | hypothetical protein (EC:5.3.4.1) (**thiol:disulfide interchange protein DsbC †**) | **6.4** | **1.5** | **4.4** |
| Swit_5370 | Type IV conjugative transfer system protein TraV | **2.6** | **1.6** | **4.2** |
| Swit_5373 | hypothetical protein | **3.9** | **1.4** | **5.3** |
| Swit_5375 | Type-F conjugative transfer system protein TraW | **16.6** | **4.1** | **4.1** |
| Swit_5378 | Type-F conjugative transfer system pilin assembly protein TrbC | **1.3** | **4.3** | **5.7** |
| Swit_5379 | Mating pair stabilisation TraN | **6.9** | **2.4** | **2.9** |
| Swit_5381 | TraF-like protein | **2.5** | **1.3** | **3.2** |
| Swit_5382 | TraH family protein | **2.0** | **1.6** | **3.3** |
| Swit_5383 | TraG domain protein | **2.1** | **1.2** | **2.6** |
