# Supplementary material for: Sphingomonas wittichii Strain RW1 Genome-Wide Gene Expression Shifts in Response to Dioxins and Clay
Source: PLoS One. 2016 Jun 16;11(6):e0157008. doi: 10.1371/journal.pone.0157008 (PMC4911050; doi:10.1371/journal.pone.0157008)
Supplement: S2 Table — Colors as in S1 Table. (DOCX) [file pone.0157008.s002.docx]

S2 Table. Genes differentially expressed between DD, and the early slow growth phase culture DD1. Colors as in S1 Table.

| **Gene ID** | **Product** | | | **Fold Change**  **DD/SUC** | **Fold Change DD1/SUC** | | **Fold Change DD1/DD** | |
| --- | --- | --- | --- | --- | --- | --- | --- | --- |
| Swit_0690 | | 40-residue YVTN family beta-propeller repeat protein | **1.2** | | | **9.7** | | **8.0** |
| Swit_4550 | | ABC transporter related | **1.5** | | | **12.8** | | **8.4** |
| Swit_0688 | | acetate--CoA ligase (EC:6.2.1.1) | **1.2** | | | **4.5** | | **5.6** |
| Swit_0703 | | aldehyde dehydrogenase (EC:1.2.1.3) | **1.8** | | | **20.9** | | **11.3** |
| Swit_2508 | | aliphatic sulfonates family ABC transporter, periplasmic ligand-binding protein | **1.2** | | | **12.3** | | **10.1** |
| Swit_2509 | | Alkanesulfonate monooxygenase (EC:1.14.14.5) | **1.4** | | | **7.7** | | **11.1** |
| Swit_4730 | | Alpha/beta hydrolase fold-3 domain protein | **2.5** | | | **7.8** | | **19.5** |
| Swit_4548 | | asparagine synthase (EC:6.3.5.4) | **2.3** | | | **20.2** | | **8.7** |
| Swit_0686 | | beta-lactamase domain protein | **1.9** | | | **15.6** | | **8.4** |
| Swit_0692 | | extracellular solute-binding protein, family 3 | **1.9** | | | **13.8** | | **7.3** |
| Swit_0145 | | Glutathione S-transferase, N-terminal domain (EC:2.5.1.18) | **2.2** | | | **16.6** | | **7.6** |
| Swit_1412 | | glutathione-dependent formaldehyde-activating, GFA | **1.9** | | | **12.8** | | **6.6** |
| Swit_4632 | | histidine ammonia-lyase (EC:4.3.1.3) | **2.8** | | | **2.0** | | **5.6** |
| Swit_3581 | | hypothetical protein | **3.8** | | | **6.7** | | **25.4** |
| Swit_3430 | | hypothetical protein | **5.4** | | | **61.6** | | **11.4** |
| Swit_2263 | | hypothetical protein | **2.5** | | | **4.2** | | **10.7** |
| Swit_0689 | | hypothetical protein | **1.3** | | | **13.0** | | **10.1** |
| Swit_3596 | | hypothetical protein | **2.9** | | | **24.1** | | **8.2** |
| Swit_4547 | | hypothetical protein | **5.1** | | | **41.0** | | **8.1** |
| Swit_4435 | | hypothetical protein | **1.3** | | | **6.3** | | **8.1** |
| Swit_2130 | | hypothetical protein | **1.1** | | | **5.6** | | **6.3** |
| Swit_4539 | | NAD-dependent epimerase/dehydratase (EC:5.1.3.2) | **2.0** | | | **15.3** | | **7.6** |
| Swit_3582 | | peptidase M19, renal dipeptidase | **1.2** | | | **5.6** | | **6.7** |
| Swit_2421 | | peptidase M61 domain protein | **1.6** | | | **6.4** | | **10.0** |
| Swit_0702 | | protein of unknown function DUF779 | **1.4** | | | **19.9** | | **14.4** |
| Swit_0684 | | pseudoazurin | **1.8** | | | **18.7** | | **10.1** |
| Swit_0693 | | Pyrrolo-quinoline quinone | **1.8** | | | **13.7** | | **7.6** |
| Swit_0697 | | short-chain dehydrogenase/reductase SDR (EC:1.1.1.47) | **2.2** | | | **13.8** | | **6.3** |
| Swit_2477 | | TonB-dependent receptor | **1.5** | | | **10.2** | | **6.8** |
| Swit_0881 | | transcriptional regulator, PadR-like family | **4.9** | | | **34.8** | | **7.1** |
| Swit_0701 | | Uncharacterized membrane-anchored-like protein | **1.3** | | | **8.3** | | **10.6** |
