# Supplementary material for: Sphingomonas wittichii Strain RW1 Genome-Wide Gene Expression Shifts in Response to Dioxins and Clay
Source: PLoS One. 2016 Jun 16;11(6):e0157008. doi: 10.1371/journal.pone.0157008 (PMC4911050; doi:10.1371/journal.pone.0157008)
Supplement: S3 Table — Down-regulated genes are marked in red background, genes without significant change are marked in tan background. (COG N: Cell motility, COG M: Cell wall/membrane/envelope biogenesis; COG O: Post-translational modification, protein turnover, chaperones; COG T: Signal transduction mechanisms; COG U: Intracellular trafficking, secretion, and vesicular transport) (DOCX) [file pone.0157008.s003.docx]

S3 Table. Expression changes of genes involved in cell motility under SAP, DD, and DF relative to SUC. Down-regulated genes are marked in red background, genes without significant change are marked in tan background. (COG N: Cell motility, COG M: Cell wall/membrane/envelope biogenesis; COG O: Post-translational modification, protein turnover, chaperones; COG T: Signal transduction mechanisms; COG U: Intracellular trafficking, secretion, and vesicular transport)

| **Gene ID** | **Product** | **COG CATEGORY** | | **SAP/SUC** | **DD/SUC** | **DF/SUC** |
| --- | --- | --- | --- | --- | --- | --- |
| Swit_0047 | YcfA family protein | | N |  |  |  |
| Swit_0213 | flagellar hook-associated 2 domain protein | | N |  |  |  |
| Swit_1259 | OmpA/MotB domain protein | | N |  |  |  |
| Swit_1260 | Flagellar motor component-like protein | | N |  |  |  |
| Swit_1261 | flagellin domain protein | | N |  |  |  |
| Swit_1262 | flagellar hook-associated protein FlgK | | N |  |  |  |
| Swit_1264 | flagellar P-ring protein | | N |  |  |  |
| Swit_1265 | flagellar L-ring protein | | N |  |  |  |
| Swit_1266 | flagellar basal-body rod protein FlgG | | N |  |  |  |
| Swit_1267 | flagellar basal-body rod protein FlgF | | N |  |  |  |
| Swit_1268 | flagellar basal body FlaE domain protein | | N |  |  |  |
| Swit_1269 | flagellar hook capping protein | | N |  |  |  |
| Swit_1270 | flagellar basal-body rod protein FlgC | | N |  |  |  |
| Swit_1271 | flagellar basal-body rod protein FlgB | | N |  |  |  |
| Swit_1272 | MotA/TolQ/ExbB proton channel | | N |  |  |  |
| Swit_1283 | flagellin domain protein | | N |  |  |  |
| Swit_1284 | flagellin domain protein | | N |  |  |  |
| Swit_1288 | flagellar motor switch protein FliG | | N |  |  |  |
| Swit_1293 | flagellar basal body-associated protein FliL | | N |  |  |  |
| Swit_1458 | flagellar motor switch protein FliM | | N |  |  |  |
| Swit_4867 | hypothetical protein | | N |  |  |  |
| Swit_1263 | Rod binding-like protein | | N, M, O |  |  |  |
| Swit_1274 | hypothetical protein | | N, O |  |  |  |
| Swit_0065 | CheA signal transduction histidine kinase (EC:2.7.13.3) | | N, T |  |  |  |
| Swit_0066 | putative CheW protein | | N, T |  |  |  |
| Swit_0068 | response regulator receiver modulated CheB methylesterase (EC:3.1.1.61) | | N, T |  |  |  |
| Swit_0069 | Protein-glutamate O-methyltransferase (EC:2.1.1.80) | | N, T |  |  |  |
| Swit_1313 | methyl-accepting chemotaxis sensory transducer | | N, T |  |  |  |
| Swit_2724 | methyl-accepting chemotaxis sensory transducer | | N, T |  |  |  |
| Swit_2932 | methyl-accepting chemotaxis sensory transducer | | N, T |  |  |  |
| Swit_3186 | response regulator receiver modulated CheB methylesterase (EC:3.1.1.61) | | N, T |  |  |  |
| Swit_3822 | methyl-accepting chemotaxis sensory transducer | | N, T |  |  |  |
| Swit_3980 | methyl-accepting chemotaxis sensory transducer | | N, T |  |  |  |
| Swit_4628 | methyl-accepting chemotaxis sensory transducer | | N, T |  |  |  |
| Swit_5354 | MCP methyltransferase, CheR-type (EC:2.1.1.80) | | N, T |  |  |  |
| Swit_5355 | CheB methylesterase (EC:3.1.1.61) | | N, T |  |  |  |
| Swit_0163 | Type IV secretory pathway TrbD component-like protein | | N, U |  |  |  |
| Swit_0214 | type III secretion exporter | | N, U |  |  |  |
| Swit_0215 | flagellar biosynthetic protein FliR | | N, U |  |  |  |
| Swit_0216 | flagellar biosynthetic protein FliQ | | N, U |  |  |  |
| Swit_0217 | flagellar biosynthetic protein FliP | | N, U |  |  |  |
| Swit_1279 | flagellar biosynthesis protein FlhA | | N, U |  |  |  |
| Swit_1286 | flagellar hook-basal body complex subunit FliE | | N, U |  |  |  |
| Swit_1287 | flagellar M-ring protein FliF | | N, U |  |  |  |
| Swit_1289 | hypothetical protein | | N, U |  |  |  |
| Swit_1290 | ATPase, FliI/YscN family (EC:3.6.3.14) | | N, U |  |  |  |
| Swit_1459 | flagellar motor switch protein FliN | | N, U |  |  |  |
| Swit_2583 | type II secretion system protein | | N, U |  |  |  |
| Swit_2584 | general secretory pathway protein E | | N, U |  |  |  |
| Swit_2587 | general secretion pathway protein G | | N, U |  |  |  |
| Swit_2591 | general secretion pathway protein D | | N, U |  |  |  |
| Swit_3510 | type II secretion system protein | | N, U |  |  |  |
| Swit_3693 | type IV secretory pathway, VirB3 family protein | | N, U |  |  |  |
| Swit_4864 | type II secretion system protein | | N, U |  |  |  |
| Swit_5001 | P-type DNA transfer ATPase VirB11 | | N, U |  |  |  |
| Swit_0212 | Flagellin-specific chaperone FliS-like protein | | N, U, O |  |  |  |
| Swit_1113 | peptidase A24A, prepilin type IV (EC:2.1.1.-,EC:3.4.23.43) | | N, U, O |  |  |  |
