# Supplementary figures and images for: Sphingomonas wittichii Strain RW1 Genome-Wide Gene Expression Shifts in Response to Dioxins and Clay
Source: PLoS One. 2016 Jun 16;11(6):e0157008. doi: 10.1371/journal.pone.0157008 (PMC4911050; doi:10.1371/journal.pone.0157008)

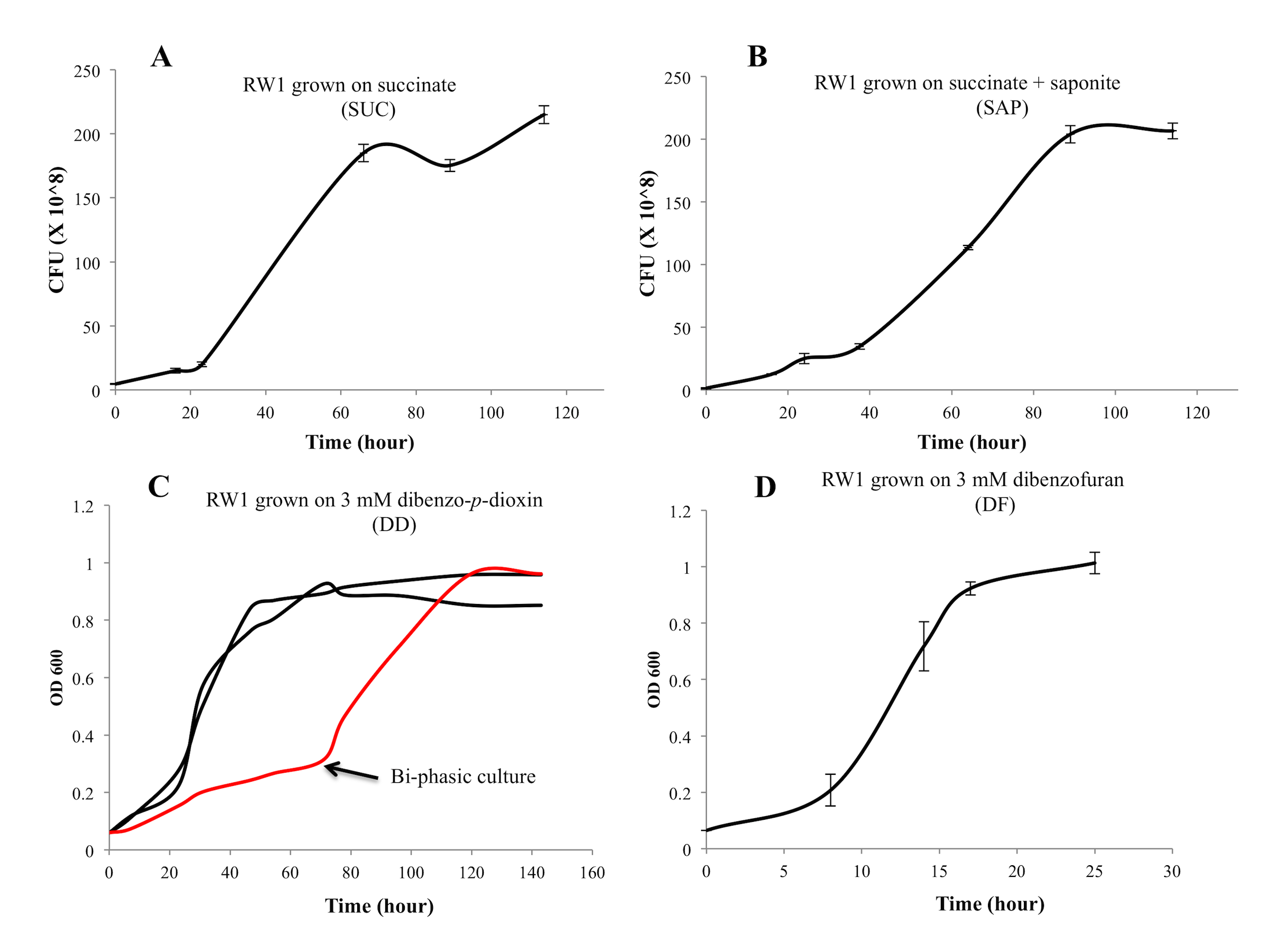

Supplement: S1 Fig — RW1 cell cultures growing on (A) succinate as sole carbon source; (B) succinate as sole carbon source and in the presence of 0.7% Cs-Saponite; (C) 3 mM dibenzo-p-dioxin as sole carbon; (D) 3 mM dibenzofuran as sole carbon source. (Note one replicate of DD had delayed, biphasic growth). (TIFF) [file pone.0157008.s004.tiff]

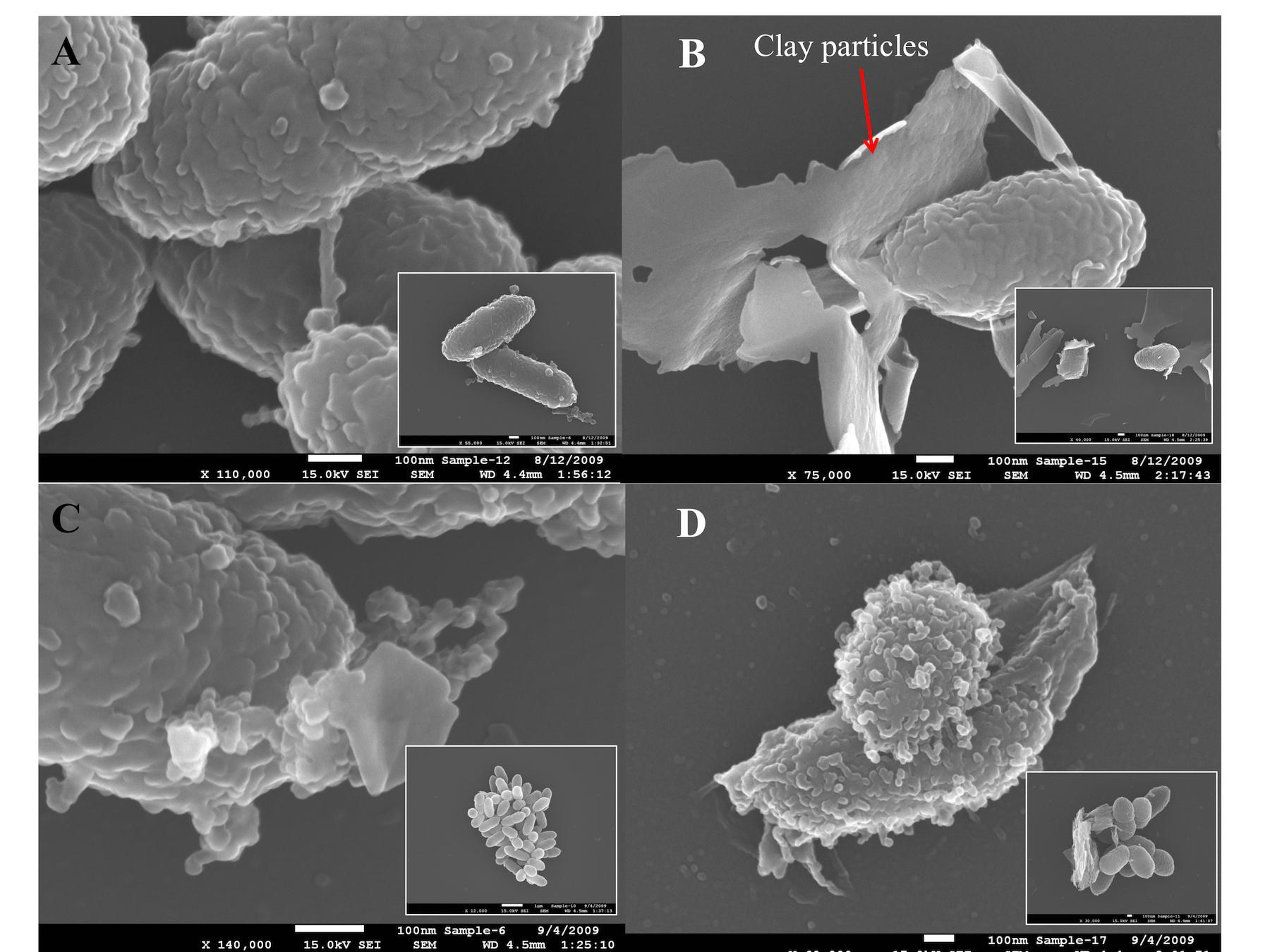

Supplement: S2 Fig — (A) SUC—cells self-aggregate, produce expolysaccharide and slime; (B) SAP—cells appear free-floating and did not aggregate or produce slime, but were attracted to smaller clay particles; (C) DD and (D) DF—cells form large aggregates, attached to DF crystals. White bar is 100 μm. Inset shows larger image of same sample. (TIFF) [file pone.0157008.s005.tiff]

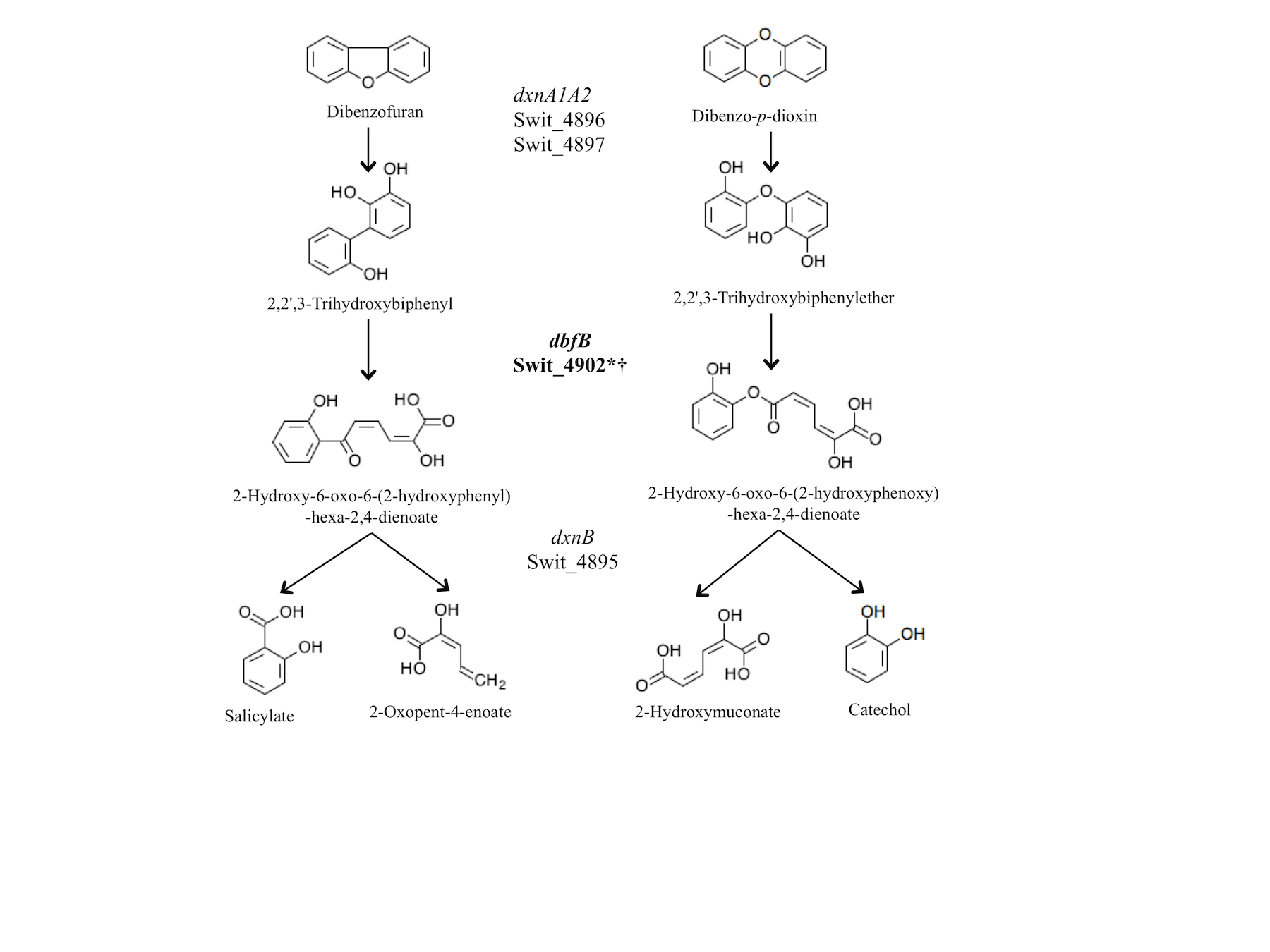

Supplement: S3 Fig — dbfB was up-regulated (*†) in response to both dibenzofuran (DF) and dibenzo-p-dioxin (DD). (TIFF) [file pone.0157008.s006.tiff]
